# BINOL-Containing Chiral Porous Polymers as Platforms for Enantiorecognition

**DOI:** 10.1021/acsami.2c18074

**Published:** 2022-11-23

**Authors:** Antonio Valverde-González, M. Carmen Borrallo-Aniceto, Mercedes Pintado-Sierra, Félix Sánchez, Avelina Arnanz, Mercedes Boronat, Marta Iglesias

**Affiliations:** † Instituto de Ciencia de Materiales de Madrid, CSIC, C/ Sor Juana Inés de la cruz, 3, Madrid 28049, Spain; ‡ Instituto de Química Orgánica General, CSIC, C/ Juan de la Cierva, 3, Madrid 28006, Spain; § Departamento de Química inorgánica, Universidad Autónoma de Madrid, Cantoblanco, Madrid 28049, Spain; ∥ Instituto de Tecnología Química, Universitat Politècnica de València- Consejo Superior de Investigaciones Científicas (UPV-CSIC), Avenida de los Naranjos s/n, 46022 Valencia, Spain

**Keywords:** chiral porous polymers, BINOL building blocks, enantioselective recognition, fluorescence, terpenes

## Abstract

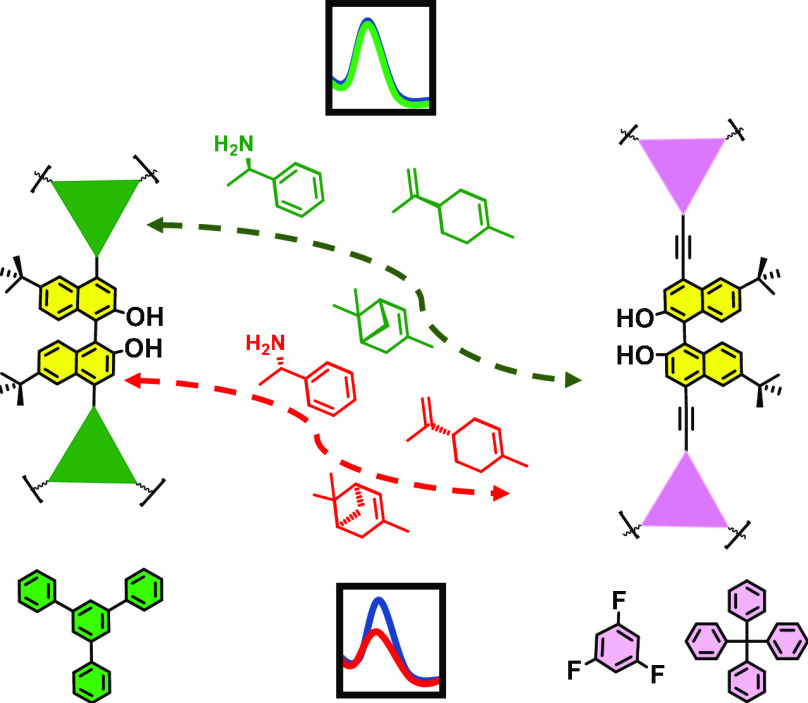

The enantioselective discrimination of racemic compounds can be achieved through the design and preparation of a new family of chiral conjugated BINOL–porous polymers (CBPPs) from enantiopure (*R*)- or (*S*)-BINOL derivatives and 1,3,5-*tris*(4-phenylboronic acid)benzene or 1,3,5-*tris*(4-ethynylphenyl)benzene, 1,3,5-triethynyl-2,4,6-trifluorobenzene, and *tetra*(4-ethynylphenyl)methane as comonomers following Suzuki–Miyaura and Sonogashira–Hagihara carbon–carbon coupling approaches. The obtained CBPPs show high thermal stability, a good specific surface area, and a robust framework and can be applied successfully in the fluorescence recognition of enantiomers of terpenes (limonene and α-pinene) and 1-phenylethylamine. Fluorescence titration of CBPPs-OH in acetonitrile shows that all Sonogashira hosts exhibit a preference for the (*R*)-enantiomer over the (*S*)-enantiomer of 1-phenylethylamine, the selectivity being much higher than that of the corresponding BINOL-based soluble system used as a reference. However, the Suzuki host reveals a preference toward (*S*)-phenylethylamine. Regarding the sensing of terpenes, only Sonogashira hosts show enantiodifferentiation with an almost total preference for the (*S*)-enantiomer of limonene and α-pinene. Based on the computational simulations and the experimental data, with 1-phenylethylamine as the analyte, chiral recognition is due to the distinctive binding affinities resulting from N···H–O hydrogen bonds and the π–π interaction between the host and the guest. However, for limonene, the geometry of the adsorption complex is mostly governed by the interaction between the hydroxyl group of the BINOL unit and the C=C bond of the iso-propenyl fragment. The synthetic strategy used to prepare CBPPs opens many possibilities to place chiral centers such as BINOL in porous polymers for different chiral applications such as enantiomer recognition.

## 1 Introduction

A significant development of chirality research topics has been achieved, and several chiral materials have been obtained and used in enantioselective sensing (important for biotechnology, medical diagnostics, analytical chemistry, pharmaceutical industries, etc.),^1−3^ enantiomeric separation,^4^ or asymmetric catalysis.^5,6^

The enantiomeric pure BINOL (1,1′-binaphth-2,2′-diol),^7−10^ its derivatives, and some axially chiral biphenols are important chiral auxiliaries and have been applied in both asymmetric catalysis^11−13^ and chiral recognition.^14−16^ As far as the catalytic behavior of BINOLs is concerned, the enantioselectivity of asymmetric reactions catalyzed by BINOLs depends on the substituents in the different positions of their naphthalene rings. On the other hand, BINOL compounds are moderate Brønsted acids^17,18^ and only lead to acceptable enantioselectivities on limited reactions.^11,19,20^ Recently, it has been reported that suitably functionalized BINOL units can be incorporated into porous organic networks that can tailor the chiral properties of molecular catalysts.^21−24^ Chiral fluorescence sensors are a class of materials whose fluorescence emission, in the best scenario, is mainly quenched in the presence of only one analyte’s enantiomer. Different types of chiral sensing have also been described, such as electrochemical sensing,^25,26^ and interestingly, very recently, an enantiomer-selective magnetization strategy has been employed to separate crystals of conglomerates composed of racemic amino acids with very high enantioselectivities.^27,28^

The typical mechanism for this chiral recognition is based on intermolecular hydrogen bonds that can be formed between the OH groups of BINOL and certain analytes such as amines, alcohols, aminoalcohols, or carboxylic acids.^14,29−31^ So as to have chiral recognition, this interaction should be preferred with only one enantiomer.

**Figure 1:**
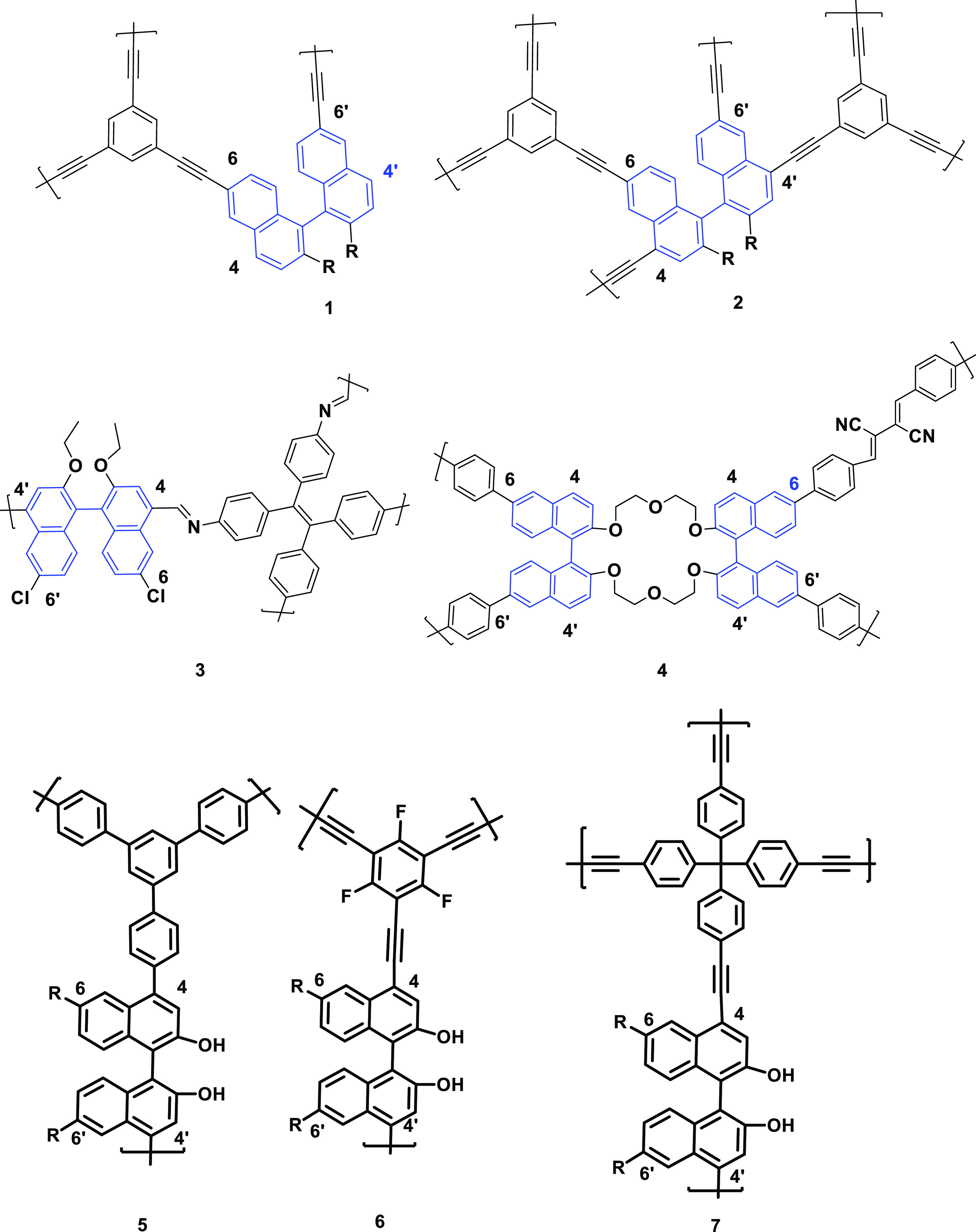
BINOL-based POPs used as sensors: 1–4 (previous work) and 5–7 (this work).

**Figure 2:**
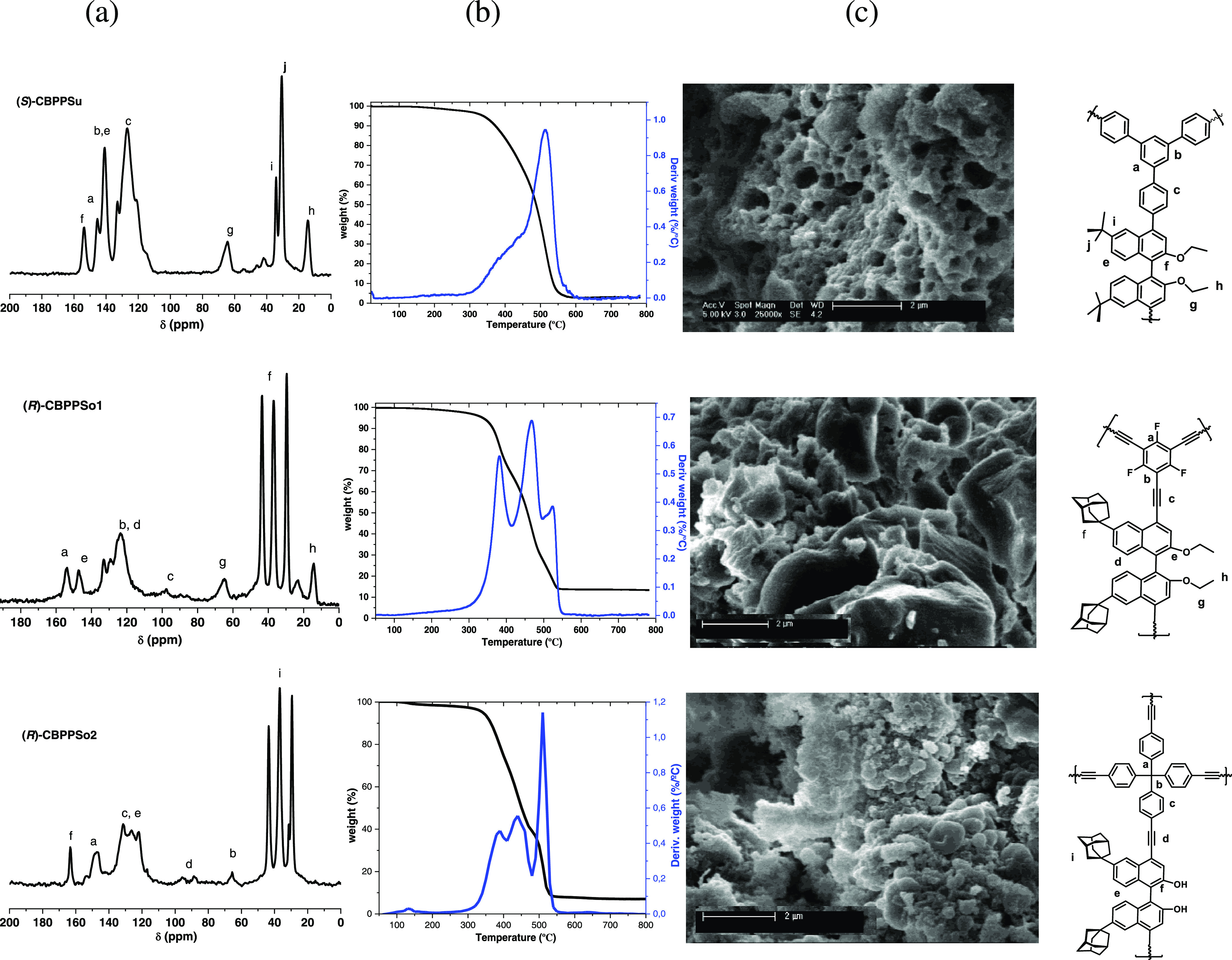
Characterization data: (a) ^13^C solid-state CP/MAS NMR spectra; (b) TGA; and (c) SEM images.

**Figure 3:**
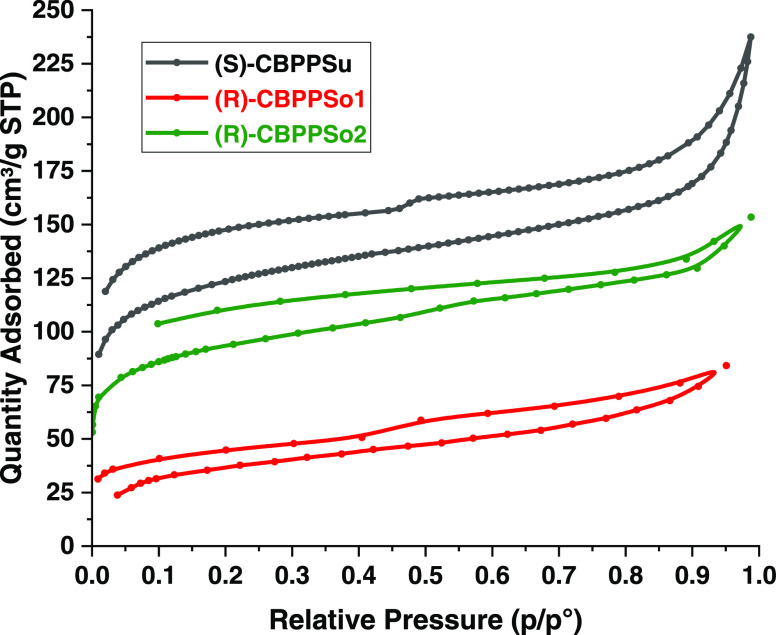
N_2_ adsorption/desorption isotherms of CBPPs-OH at 77 K.

**Figure 4:**
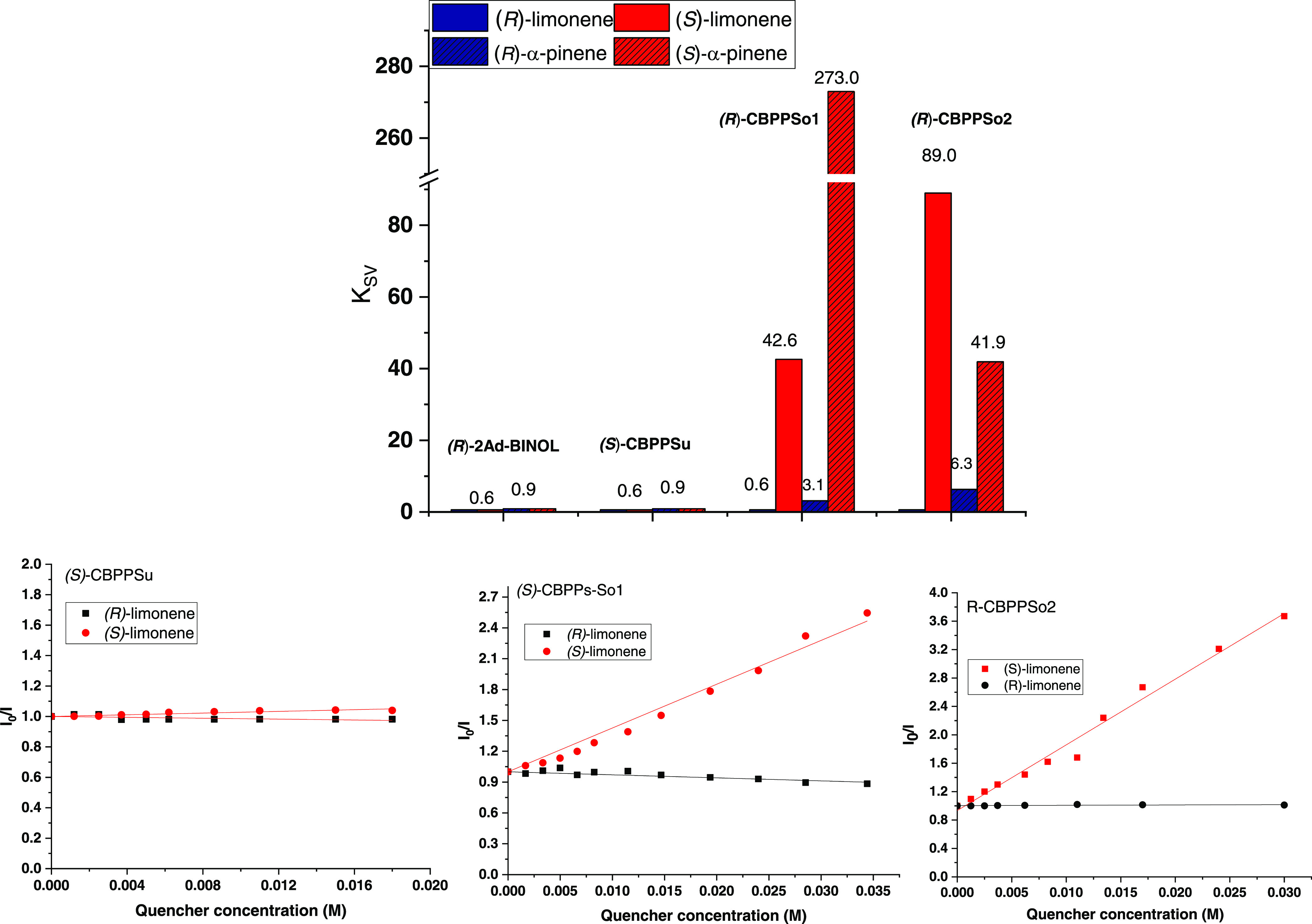
(Top) Estimated *K*_SV_ values obtained by fluorescence quenching analysis with limonene: CBPP (1.0 mg), MeCN (4 mL), and quencher (0.5 M). (Down) Stern–Völmer plots of CBPPs upon titration (see SI).

Porous organic polymers (POPs) have been developed by materials chemists in the last few years.^32−34^ POPs can be prepared from singular organic building blocks using Suzuki and Sonogashira–Hagihara C–C couplings (polymeric aromatic frameworks, PAFs, conjugated porous polymers, CMPs),^35−38^ reversible condensations (crystalline covalent organic frameworks, COFs),^39^ or Friedel–Crafts reactions (triazine based frameworks,^40−42^ hyper-cross-linked polymers, HCPs).^43,44^ The properties of POPs such as exceptional specific surface area and versatility of structural design are beneficial for different applications (chemical reactions, sensing properties, etc.) because of the homogeneous distribution of the active sites; the application of POPs as chiral sensors implies the use of chiral skeletons that generate chiral microenvironments that can enhance chiral discrimination.^3,15,45,46^ Some examples where POPs have been used as chiral sensors include BINOL,^24,47^ dibinaphthyl-22-crown-6,^48^ or 1,3,5-triformylphloroglucinol^49^ as structural units (Figures 1, 1–4).

**Figure 5:**
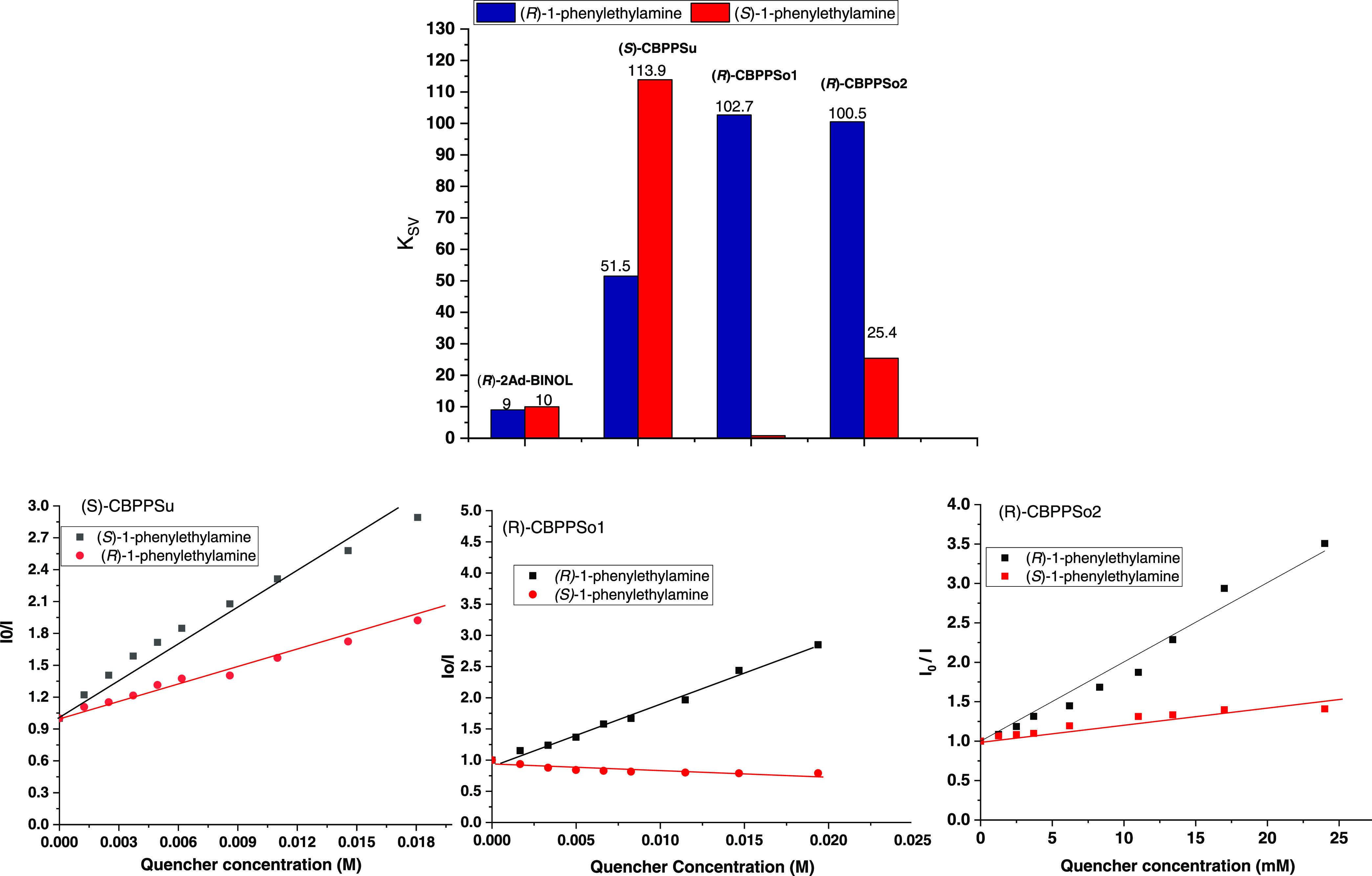
(Top) Estimated *K*_SV_ values obtained by fluorescence quenching analysis with 1-phenylethylamine: CBPP (1.0 mg), MeCN (4 mL), and quencher (0.5 M). (Down) Stern–Völmer plots of CBPPs upon titration (see SI).

**Figure 6:**
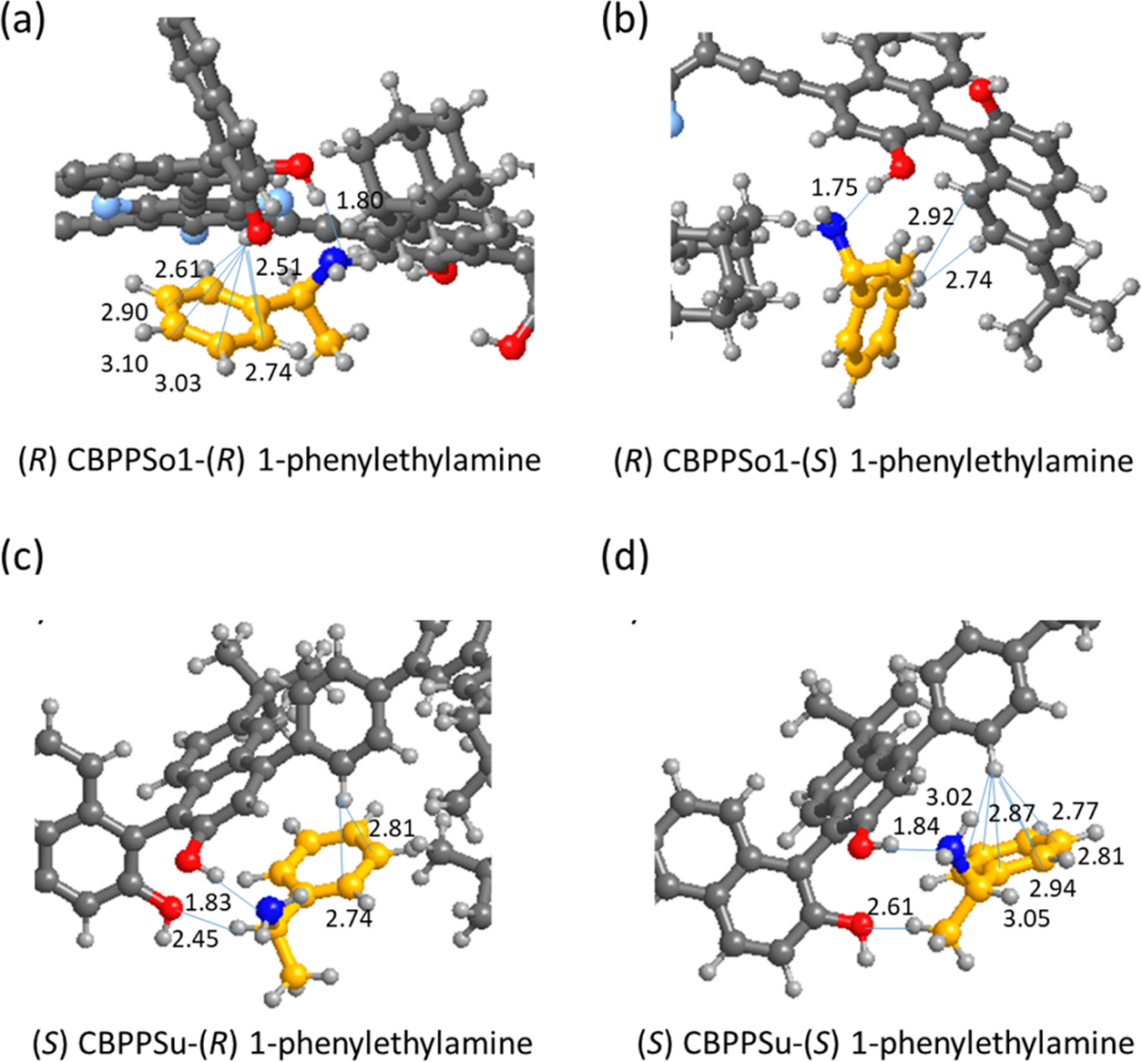
Optimized geometry of 1-phenylethylamine (in yellow) interacting with (*R*)-CBPPSo1 (a, b) and (*S*)-CBPPSu (c,d) polymers. Optimized bond lengths in angstrom. C: gray; H: white: O: red; N: blue, F: light blue. The inset in panels (b, c) shows the relative orientation of the BINOL and 1-phenylethylamine aromatic rings.

Herein, we report a series of new chiral conjugated porous polymers containing substituted BINOLs and different aromatic units in the framework (named CBPPs-OH) prepared through different polymerization strategies to evaluate the synthetic influence on properties such as the BET surface or the availability of active centers (Figures 1, 5–7). Their chiral recognition performance was evaluated for (*R*)- and (*S*)-enantiomers of limonene, α-pinene, and 1-phenylethylamine. High enantiodiscrimination was observed, showing that CBPPs could be utilized as chiral solid fluorescent sensors. We have also done computational simulations that shed light on the structure–enantiodiscrimination relationship.

## 2 Experimental Section

### 2.1 Preparation of BINOL Polymers (CBPPs-OEt)

The experimental details of the synthesis of the monomers are provided in the Supporting Information.

#### 2.1.1 Suzuki–Miyaura Coupling

**Scheme 1:**
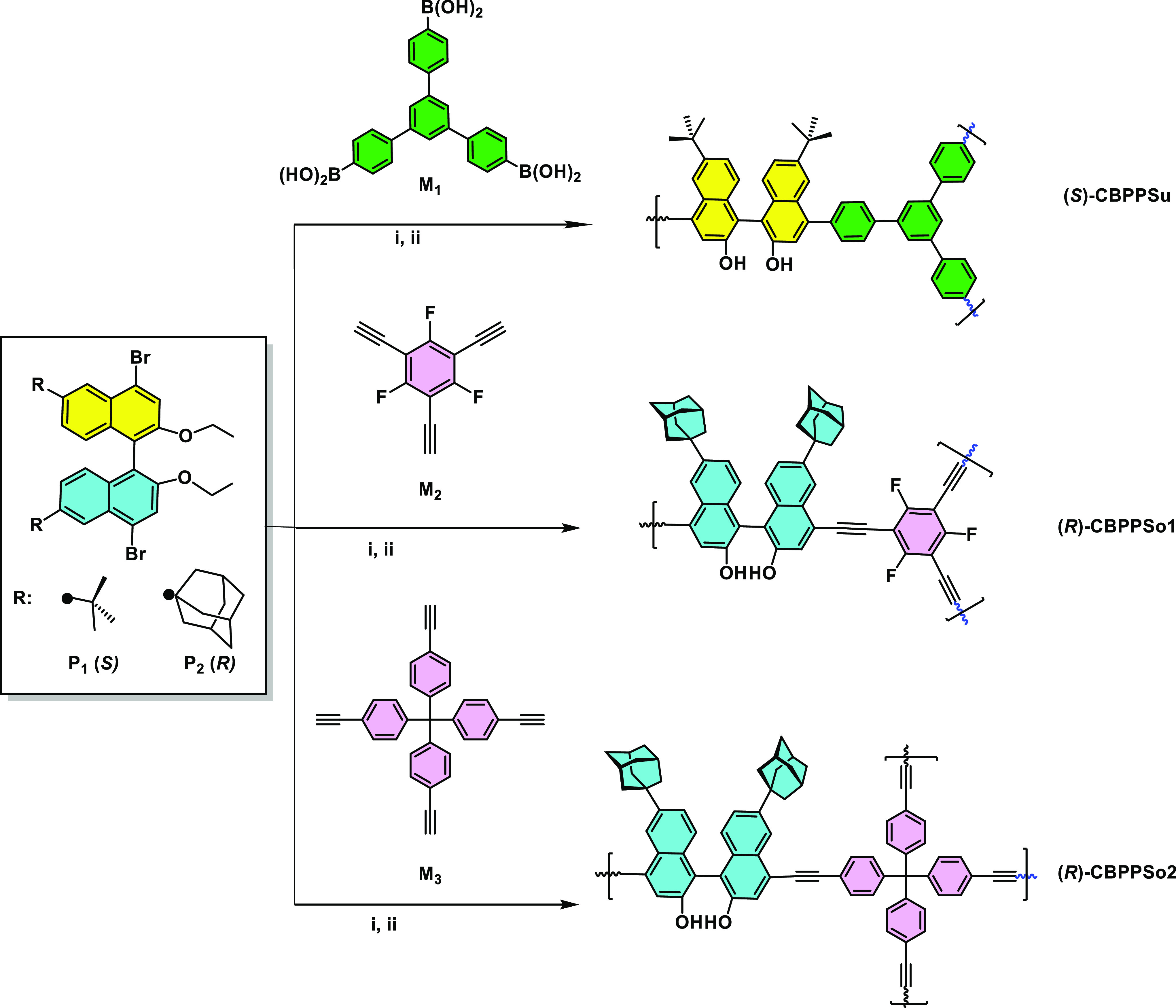
Synthetic Routes to CBPPs-OH. (i) Catalyst Pd(dppf)_2_Cl_2_ (for Further Synthetic Details, see the SI) and (ii) O-Deprotection (BBr_3_)

General method: BINOL monomer **P1** (Scheme 1) (0.326 mmol, 1.5 equiv), 1,3,5-triphenylbenzene-4′,4″,4‴-triboronic acid (**M1**, Scheme 1)^50^ (95.5 mg, 0.218 mmol, 1.0 equiv), K_2_CO_3_ (2 mL, 2 M, 4.20 equiv), and dry tetrahydrofuran (THF) (4.5 mL) were introduced in a sealed tube and deaerated with argon for 15 min. After that, catalyst Pd(dppf)Cl_2_ (1.1 mg, 9.8 μmol, 3%) was added. The reaction was stirred overnight at 100 °C. The resulting solid was filtered and thoroughly washed with H_2_O. The solid was stirred with a mixture of acetone–H_2_O and KCN overnight to remove the Pd(0) residues. Then, it was filtered and dried to obtain the final product.

#### 2.1.2 Sonogashira–Hagihara Coupling

General method: The polymer was prepared following a similar procedure to that used for the Suzuki type. Thus, BINOL monomers (**P2**, Scheme 1) (40 mg, 0.052 mmol, 1.0 equiv), alkyne (**M2** or **M3**, Scheme 1, 0.5 equiv), 2 mL of diisopropylamine (DIPA), and dry dimethylformamide (DMF) (4.0 mL) were introduced in a sealed tube and deaerated with argon for 15 min. After that, catalyst Pd(PPh_3_)_4_ (9.8 μmol, 3%) and CuI (5 μmol, 1.5%) were added. The reaction mixture was stirred overnight at 100 °C. The resulting solid was filtered and thoroughly washed with DMF and H_2_O and finally stirred overnight in a mixture of acetone–H_2_O with KCN to remove the remaining Pd(0) species. Then, it was filtered and dried at 100 °C under vacuum to obtain the final product.

#### 2.1.3 Deprotection of CBPPs-OEt (CBPPs-OH)

General deprotection procedure: An excess of BBr_3_ in dichloromethane (10 mL per 100 mg of polymer) was added at −78 °C to a suspension of the CBPP and stirred for 2 h; then, the mixture was heated to room temperature and stirred for two days at the said temperature. To quench the reaction, a saturated aqueous solution of NaHCO_3_ (5 mL) was added, and the mixture was stirred for 2 h; the resulting polymer was filtered and exhaustively washed with water. Subsequently, the polymer was stirred in warm methanol for an additional two hours. Finally, the solid is filtered and washed with methanol, acetone, and diethyl ether.

### 2.2 Procedure for Quenching Measurements

Polymers were soaked in deoxygenated acetonitrile (MeCN) to remove any solvent remaining in the porous polymers. Then, the solids were dried under vacuum and mechanically ground with an agate mortar and pestle. Exactly 1.0 mg of the corresponding CBPPs-OH was placed in a quartz cuvette with 4 mL of MeCN, leading to a cloudy dark suspension. Moreover, 0.5 M enantiopure solutions of quenchers were prepared.

### 2.3 Models and Methods

All calculations are based on density functional theory (DFT) and are performed with Gaussian09 software^51^ using the M062X functional^52^ and the 6–311g(d,p) basis set for O, C, N, F, and H atoms.^53^ The models employed in the simulations contain one central structural building block (**M1** or **M2**) linked to two chiral BINOL building blocks substituted with *tert*-butyl or adamantine. The (*R*) and (*S*) enantiomers of the two analytes investigated theoretically, limonene and 1-phenylethylamine, were placed with different initial orientations in different locations, either close to the hydroxyl groups or in the region between the two chiral BINOL units. The geometry of all of the resulting systems was fully optimized without restrictions, and the most stable structures obtained are discussed in the manuscript.

## 3 Results and Discussion

The BINOL skeleton was the building block of choice to incorporate into conjugated porous polymers because of its structural rigidity, which can contribute to the assembly of the rigid PAF networks (CBPPs-OH). We have applied two strategies to prepare the polymers: (a) Suzuki–Miyaura and (b) Sonogashira–Hagihara cross-couplings (Scheme 1). Precursor P1 was synthesized in three steps starting from (*S*)-1,1′-binaphthol and *tert*-butyl chloride in dichloromethane and AlCl_3_ at −78 °C,^54^ protection with iodoethane in MeCN, and subsequent bromination (Scheme S2). Besides, P2 was obtained from 1-adamantanol and (*R*)-1,1′-binaphthol as reported.^55^ The Suzuki polymer was obtained from the reaction of (*S*)-4,4′-dibromo-2,2′-diethoxy-6,6′-*tert*butyl-BINOL (precursor P1) as a chiral building block and 1,3,5-*tris*(4-phenylboronic acid)benzene (M1) as a structural building block in the presence of Pd(dppf)_2_Cl_2_ as the catalyst (Scheme 1). Sonogashira-type BINOL frameworks were synthesized from P1 (*S*-enantiomer) or P2 (*R*-enantiomer) as chiral building blocks and 1,3,5-triethynyl-2,4,6-trifluorobenzene (M2) or *tetra*((4-ethynylphenyl)methane) (M3) as structural building blocks using CuI and Pd(PPh_3_)_4_ as the catalyst (Scheme 1). The isolated solids were exhaustively washed and palladium residues were removed by treating them with KCN in a mixture of acetone and water. Treatment of ethoxy-derivatives with BBr_3_ in CH_2_Cl_2_ gives the corresponding OH derivative. The frameworks were obtained in quantitative yields and are insoluble in water and in all of the most common organic solvents.

To confirm that monomers are part of the polymer network, we have recorded the ^13^C solid-state CP/MAS NMR spectra of the protected CBPPs-OEt materials (Figure 2a). As can be observed, the chemical shifts at ∼160 to 150 ppm correspond to the C–O bonds in BINOL, and the peaks at 150–110 ppm correspond to the aromatic carbons. The signals from ethoxy groups can be appreciated at 64 and 15 ppm. The aliphatic carbons corresponding to *tert*-butyl (at 30 and 35 ppm) or adamantine groups (at 30, 35, and 45 ppm) can be easily observed. The ^13^C-NMR spectra of Sonogashira derivatives also show two weak signals at 95 and 85 ppm corresponding to the C≡C spacer.

To evaluate the thermal stability of polymers, we have performed thermogravimetric analysis (TGA, Figure 2b). The TGA curves exhibited that all polymers are stable up to 400 °C, indicating their good thermal stability. X-ray diffraction indicates that all polymers are amorphous. Scanning electron microscopy (SEM) images showed that these materials (Figure 2c) displayed spherical, irregular surfaces with hierarchical porosity.

To check if the deprotection reaction of CBPPs-OEt with BBr_3_ is complete, we have recorded the FT-IR spectra (Figure S3). Thus, the free OH bands after deprotection of the BINOL moiety can be observed at 3533–3547 cm^–1^, and the vibration frequency peaks at ∼3050 cm^–1^ are assigned to the aromatic C–H, whereas intense aliphatic ν_CH_ stretching bands appear at the region of 2900–2845 cm^–1^ due to the adamantyl or *tert*-butyl groups. In addition, the IR spectra of CBPPs materials exhibit strong absorptions around 810 cm^–1^ due to C–H out-of-plane bending vibrations. Sonogashira polymers also showed a weak band at 2188–2203 cm^–1^ due to the alkyne spacer.

The porosity and calculated surface areas (Brunauer–Emmett–Teller (BET)) of networks were studied by the analysis of nitrogen sorption isotherm curves obtained at 77 K (Table 1). Figure 3 shows the corresponding isotherms (type I with hysteresis loops, according to the IUPAC classification^56^), indicating that micro- and mesopores coexist in the materials.^47^ As can be seen (Table 1, Figure 3), Suzuki polymer (*S)*-CBPPSu has a *S*_BET_ of 456 m^2^ g^–1^, which is higher than previously reported 365 m^2^ g^–1^ for (*R*)*-*CBPPSu,^21^ which indicates that substitution at 6,6’ positions has little effect on porosity. Sonogashira C–C coupling polymer (*R*)-CBPPSo1 (with the smallest colinker, M2) exhibits lower BET surfaces (131 m^2^ g^–1^). Pore size distributions (calculated by density functional theory) shown in Figure S4 suggest that the majority of the porosity is in the micropore regime. A minor proportion is in the mesopore and macropore regions.

**Table 1:** Porous Properties of Polymers

entry	material	*S*_BET_ (m^2^ g^–1^)^a^	*V* (cm^3^ g^–1^)^b^	pore size (nm)
1	(*S*)-CBPPSu	456	0.33	2.92
2	(*R*)-CBPPSo1	131	0.11	1.23
3	(*R*)-CBPPSo2	343	0.19	1.93

a At *P*/*P*_o_: 0.99.

b Calculated from the nitrogen adsorption isotherm.

### 3.1 Enantioselective Recognition

Enantiomeric sensing is probably one of the most challenging types of chemical sensing, being important in areas such as biotechnology, medical diagnostics, or for the synthesis of biologically significant molecules for fragrances, agrochemical, pharmaceutical, and food additives industries.^15^ Homochiral porous materials such as MOFs, COFs, etc. are promising for efficient chiral resolution, which display excellent properties for chiral separation applications,^16^ the enantioselective recognition usually coming from a particular host–guest interaction between chiral analytes and the chiral framework.^57^ This interaction can lead to changes in NMR, circular dichroism, or fluorescence spectra, in particular structures with OH- or NH-groups that can benefit the interactions.^58^ It has been reported that when BINOL was introduced into the frameworks of MOFs or COFs, the chiral discrimination of mainly amino alcohol was improved.^24,59^ CBPPs-OH have optical functional groups accessible to guest compounds, which may favor their use as chiral fluorescence sensors. Optical spectroscopy has been extensively used in chiral recognition due to advantages such as high sensitivity and low cost; thus, enantioselective sensing was studied by exploring the optical spectra upon the analyte–network interaction process. Before the sensing study, the photophysical characteristics of CBPPs were studied by UV–visible and fluorescence spectroscopy. CBPPs-OH polymers crushed in a mortar and suspended in MeCN show in their UV–vis spectra absorption maxima at 228, 278 nm ((*S*)-CBPPSu), 224, 272 nm ((*R*)-CBPPSo1), and 224, 280 nm ((*R*)-CBPPSo2) (Figure S5 in ESI). These bands are due to π–π*, as has been reported for other BINOL derivatives.^60^ To confirm that the emissions observed are due to the fluorescence phenomena, the same spectra were recorded at λ_ex_ of choice ±10 nm; if the maximum λ_em_ is maintained, we are observing fluorescence emission. All CBPPs are fluorescent with emission maxima at 423 nm ((*S*)-CBPPSu), 372 nm ((*R*)-CBPPSo1), and 337 nm ((*R*)-CBPPSo2) excited by λ = 278, 272, and 280 nm, respectively, redshifted compared to that of soluble reference (*R*)-2Ad-BINOL (λ_ex_ = 314 nm, λ_em_ = 363 nm) (Figure S5), which indicates that the polymer frameworks allow an extension of π-electronic conjugation with respect to the building units except (*R*)-CBPPSo2. These emission bands are in good agreement with other BINOL-derived systems (emission band at 350–450 nm).^14^

The enantioselective fluorescence recognition of CBPPs-OH was studied by the addition of chiral analytes to different samples of polymers. As analytes, we have selected the two enantiomers of limonene, α-pinene, and 1-phenylethylamine. To do this, the same conditions as before were used: polymers (1 mg) were suspended in MeCN (4 mL); subsequently, aliquots containing different amounts (at mM concentrations) of one enantiomer of the analytes were added to the suspensions, and the fluorescence emission spectra were recorded (details can be found in the Supporting Information).

Chiral sensing of terpenes such as α, β-pinenes or limonene is a major challenge for molecular recognition.^61^ So, we started our sensing studies with (*R*)- and (*S*)-limonene as analytes (Table 2, Figures 4 and S7–S10). Sonogashira-type polymers (CBPPSo) showed different fluorescence quenching based on the added enantiomer; however, the Suzuki-type polymer is not able to discriminate between the two enantiomers. The fluorescence band of (*R*)-2Ad-BINOL, used as a homogeneous control, does not change in the presence of any limonene enantiomers. The fluorescence quenching efficiency is determined by monitoring changes in the fluorescence band and is associated with the Stern–Volmer constant (*K*_SV_),^62^ as can be seen in Table 2 and Figure 4. When (*R*)-CBPPSo1 is the host, *K*_SV_ for (*S*)-limonene is 42.6 and *K*_SV_ for (*R*)-limonene cannot be determined and is assumed to be virtually zero since the irregular and little fluorescence quenching does not follow the Stern–Volmer equation. If (*R*)-CBPPSo2 is the host, *K*_SV_ for the (*S*)-isomer is 89.0 M^–1^ (0.6 M^–1^ for (*R*)-limonene), giving a *Q*_R_ (*K*_SV_(major)/*K*_SV_(minor)) of 148.3.

**Table 2:**
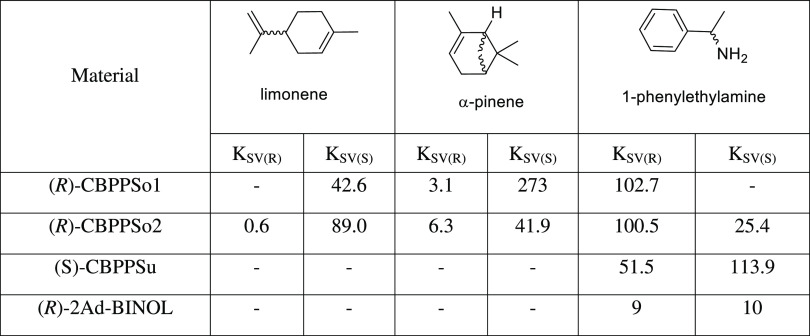
Stern–Volmer Constants *K*_SV_ (M^–1^) of CBPPs-OH with Different Analytes^a^

a Obtained from three experiments at 298 K, CBPP (1.0 mg), MeCN (4 mL), and quencher (0.5 M); estimated errors are <5%.

A similar behavior was observed when (*R*)- and (*S*)-α-pinene were employed as chiral analytes; the changes in the fluorescence band are only observed for the (*S*)-isomer (Figures 4 and S11–S13). In this case, (*S*)-α-pinene quenched the fluorescence of (*R*)-CBPPSo1 and (*R*)-CBPPSo2. Again, as in the case of limonene, the Suzuki polymer does not interact with any enantiomer of α-pinene. With (*R*)-CBPPSo1 as the host, the estimated *K*_SV_ value for (*S*)-α-pinene is 273 M^–1^ (3.1 M^–1^ for (*R*)-α-pinene), affording a *Q*_R_ of 88.1. When (*R*)-CBPPSo2 is the host, the *K*_SV_ value for (*S*)-α-pinene is 41.9 M^–1^ (6.3 M^–1^ (*R*)-α-pinene), giving a *Q*_R_ of 6.7.

Besides, further sensing studies were performed with 1-phenylethylamine as the analyte (Table 2 and Figures 5 and S14–S17). Now, total enantiodiscrimination is observed with (*R*)-CBPPSo1, and (*R*)-1-phenylethylamine seems to interact with it. A notable 4.0 *Q*_R_ value is also obtained with (*R*)-CBPPSo2. In this case, (*S*)-CBPPSu also behaves as an effective chiral sensor with a *Q*_R_ value of 2.2; however, inverted enantiodiscrimination was observed with a higher decrease of the fluorescence band for the (*S*)-isomer. Also, the fluorescence of (*R*)-2Ad-BINOL used as a control showed low enantiodiscrimination with a *Q*_R_ of 1.1. The fluorescence quenching in the presence of 1-phenylethylamine could be explained mainly through a hydrogen-bonded interaction between hydroxyl groups and the amine unit, as has been previously reported^63−65^ and confirmed by the computational simulations described below. In all cases, the binaphthyl chirality (*S*) or (*R*) governs the preference toward the analyte’s enantiomer.

These results indicate that CBPPs display high enantioselective fluorescence performance toward chiral terpenes and 1-phenylethylamine. The chirality of (*R*)- and (*S*)-BINOL comes from the limited rotation of the naphthalene rings. In general, the structure of binaphthyl units with *C*_2_ symmetry is very important in the chiral induction, and the dihedral angle between the naphthalenes is controlled by the substituents at different positions (mainly at 3,3’). Besides, it has been reported that the confinement effect and chiral binding centers have an important influence on the chiral sensing capacity of different BINOL materials.^59,66^ To further evaluate the recognition properties of CBPPs toward terpenes and 1-phenylethylamine, computational simulations have been done. We have studied the interactions between the analytes and (*R*)-CBPPSo1 and (*S*)-CBPPSu to analyze the effect of the phenyl or alkyne groups of the polymers obtained via Suzuki or Sonogashira. Figures S18–S20 illustrate the most stable binding sites of the enantiomers of 1-phenylethylamine and limonene in CBPPs, respectively, and Table 3 summarizes the calculated binding energies and optimized geometries. The two enantiomers of both analytes are always found in the microenvironment generated near the BINOL units, the structural orientation of the (*R*)-enantiomer at the binding site being different from that of the (*S*)-enantiomer.

**Table 3:** Calculated Interaction Energies between CBPPs-OH Models and Different Analytes and Optimized Values of *r*(H-N) in 1-Phenylethylamine and *r*(H–C) in Limonene Complexes

	1-phenylethylamine		limonene	
material	*E*_int(R)_ (kJ mol^–1^)	*E*_int(S)_ (kJ mol^–1^)	*E*_int(R)_ (kJ mol^–1^)	*E*_int(S)_ (kJ mol^–1^)
(*R*)-CBPPSo1	–102	–78	–51	–80
(*S*)-CBPPSu	–82	–88	–56	–58
	***r*(H-N)**_**(R)**_ **(Å)**	**r(H-N)**_**(S)**_ **(Å)**	**r(H–C)**_**(R)**_ **(Å)**	**r(H–C)**_**(S)**_ **(Å)**
(*R*)-CBPPSo1	1.804	1.750	2.332	2.641
(*S*)-CBPPSu	1.835	1.844	2.347	2.356

For 1-phenylethylamine, the strong hydrogen bond between the N atom of the amino group and the proton of one hydroxyl group of the BINOL unit determines the molecular orientation and the possibility of additional interactions, such as π–π interactions between the aromatic rings or hydrogen bonds with the neighboring hydroxyl groups, as illustrated in Figure 6. Thus, the larger binding energy calculated for the (*R*)-enantiomer of 1-phenylethylamine with (*R*)-CBPPSo1 (−102 kJ mol^–1^, see Table 3) is due to the interaction of its aromatic ring with the proton of the neighboring free hydroxyl group, with six optimized H–C distances between 2.51 and 3.10 Å (Figure 5a,b). In contrast, the orientation of the (*S*)-enantiomer bonded to (*R*)-CBPPSo1 only allows two additional H–C interactions at 2.74 and 2.92 Å (Figure 6b), resulting in a less negative binding energy value of −78 kJ mol^–1^. There are multiple π–π interactions between the aromatic rings of the BINOL unit and of both (*R*)- and (*S*)-enantiomers of 1-phenylethylamine, with six intermolecular C–C distances in the 3.3–3.6 Å range. But an additional interaction between the ring and the proton of the neighboring free hydroxyl group is allowed only for the (*R*)-enantiomer, with an optimized H–C distance of 2.74 Å (see Figure 6a), leading to a preferential stabilization of this enantiomer.

The linker used to synthesize the (*S*)-CBPPSu polymer contains an additional aromatic ring that leads to a different type of interaction with 1-phenylethylamine and reverses the order of stability of (*R*)- and (*S*)-enantiomers. Besides the hydrogen bond between the N atom and one hydroxyl group of the BINOL unit, the O atom of the second hydroxyl group interacts with either the H or the methyl group of 1-phenylethylamine (see Figure 6c,d), leaving the aromatic ring of 1-phenylethylamine oriented toward the aromatic ring of the linker. In this situation, the (*S*)-enantiomer is able to form six H–C bonds with optimized distances between 2.77 and 3.05 Å, while only two of such stabilizing bonds at 2.74 and 2.81 Å are formed in the case of the (*R*)-enantiomer, resulting in slightly different binding energies (−88 and −82 kJ mol^–1^ for (*S*) and (*R*), respectively, see Table 3).

For limonene, the geometry of the adsorption complex is mostly governed by the interaction between the hydroxyl group of the BINOL unit and the C=C bond of the iso-propenyl fragment. Since the geometry of such an interaction is not so tight, additional interactions between the H atoms of the limonene ring and the C≡C bonds or aromatic rings of the CBPPs are allowed in all cases (see Figure S20), leading to smaller differences in the calculated binding energies. Nevertheless, in the Sonogashira polymer ((*R*)-CBPPSo1), the binding energies calculated for the (*S*)-enantiomer are always larger (−80 kJ mol^–1^) than those obtained for the (*R*)-enantiomer (−51 kJ mol^–1^), indicating a stronger affinity between the corresponding CBPP and the (*S*)-enantiomer, in agreement with the chiral discrimination reported in Table 2. However, for the (*S*)-CBPPSu polymer, the binding energies obtained for the (*R*) and (*S*)-enantiomers are similar (−56 and −58 kJ mol^–1^), in agreement with the absence of the interaction observed experimentally.

## 4 Conclusions

We report here a family of chiral organic polymers built from (*R*)- or (*S*)-4,4’-dibromo-2,2’-diethoxy-6,6’-substituted BINOL units and different alkynes or boronic acids as comonomers. These polymers are highly stable and have good surface BET areas (up to 516 m^2^ g^–1^). Besides, these CBPPs result in effective chiral recognition for (*R*)- or (*S*)-enantiomers of limonene, α-pinene, and 1-phenylethylamine, leading to an important higher enantiomeric recognition than the soluble (*R*)-2Ad-BINOL used as a reference, which indicates that these porous frameworks with extended π-conjugation and confinement effects are an excellent platform for the enantioselective recognition of chiral compounds. A study of the chiral recognition capabilities in MeCN reveals that all Sonogashira hosts exhibit a preference for the (*S*)-enantiomer over the (*R*)-enantiomer of terpenes and the (*R*)-enantiomer over the (*S*)-enantiomer of 1-phenylethylamine guests. The Suzuki hosts does not have chiral recognition over terpenes and shows a preference for the (*S*)-enantiomer over the (*R*)-enantiomer of the 1-phenylethylamine guest.

We can conclude that the different nature of the 4,4′-functionalization of the binaphthyl backbone (phenyl groups in Suzuki or alkyne functionalities in Sonogashira polymers) plays a significant role in the enantiodifferentiation of the analytes tested. Thus, Sonogashira hosts exhibit a preference for (*R*)-enantiomer over the (*S*)-enantiomer of 1-phenylethylamine guests, whereas the Suzuki polymer (*S*)-CBPPSu shows a preference for the (*S*)-enantiomer over the (*R*)-enantiomer of the 1-phenylethylamine guest. However, when terpenes are employed, different types of intermolecular interactions are established, the presence of an alkyne bond seems to affect the preferential molecular recognition, and the (*S*)-limonene shows higher affinity, which is in agreement with the experimental observation that only (*S*)-terpenes interact with the Sonogashira materials. These CBPPs hosts represent a very promising structure–activity relationship for the recognition of chiral analytes, especially terpenes.

## Supporting Information Available

Experimental procedures, spectroscopical characterization, and analytical data (NMR (^1^H, ^13^C), FT-IR, TGA, etc.) for materials and reaction products (PDF)
