# Supplementary material for: BINOL-Containing Chiral Porous Polymers as Platforms for Enantiorecognition
Source: ACS Appl Mater Interfaces. 2022 Nov 23;14(48):53936–46. doi: 10.1021/acsami.2c18074 (PMC10471007; doi:10.1021/acsami.2c18074)
Supplement: Supplementary file 1 — am2c18074_si_001.pdf [file am2c18074_si_001.pdf]

Supporting Information

BINOL-containing Chiral Porous Polymers as Platforms for Enantiorecognition

Antonio Valverde-González,[†§] M.Carmen Borrallo-Aniceto,[†] Mercedes Pintado-Sierra,[‡] Félix Sánchez,[‡] Avelina Arnanz,[$,*] Mercedes Boronat,[£,*] Marta Iglesias[†,*].

[†]Instituto de Ciencia de Materiales de Madrid. CSIC. C/ Sor Juana Inés de la cruz, 3. Madrid, Spain.
[‡]Instituto de Química Orgánica General. CSIC. C/ Juan de la Cierva, 3 Madrid 28006, Spain.
[$]Departamento de Química inorgánica. Universidad Autónoma de Madrid. Cantoblanco. Madrid 28049, Spain
[£]Instituto de Tecnología Química. Universitat Politècnica de València- Consejo Superior de Investigaciones Científicas (UPV-CSIC). Avda. de los Naranjos s/n, 46022 Valencia, Spain.
[§]Present address: Sorbonne Université, CNRS, Institut Parisien de Chimie Moléculaire, Equipe Chimie des Polymères, 4 Place Jussieu, 75005 Paris, France.

avi.arnanz@uam.es; boronat@itq.upv.es; marta.iglesias@icmm.csic.es

TABLE of CONTENTS

1.-Materials and Characterization Methods S3

2.-Preparation and characterization of BINOL monomers S4-S6

Scheme S1. Synthetic route to obtain monomer **P1** S4

Scheme S2. Synthetic route to obtain monomer **P2** S4

Figure S1. ^{1}H-NMR and ^{13}C-NMR of **A'** S5

Figure S2. ^{1}H-NMR and ^{13}C-NMR of precursor **P2** S6

3.- Preparation of BINOL-based polymers (CBPPs) S7

4.- Characterization of CBPPs S8

Figure S3. FT-IR spectra S8

Figure S4. Pore distribution by DFT method S8

5.- Uv-vis and fluorescence emission spectra of CBPPs. **Figure S5** S9

6.- UV-vis absorption spectra of chiral analytes. **Figure S6** S10

7.- General procedures and results for fluorescence sensing S11-S16

Figure S7-S10. Fluorescence quenching upon titration with limonene S12-S13

Figure S11-S13. Fluorescence quenching upon titration with α-Pinene S14

Figure S14-S17. Fluorescence quenching upon titration with 1-phenylethylamine S15-S16

8.- Computational Simulations S17-S19

Figure S18. Binding sites of 1-phenylethylamine in CBPPs S17

Figure S19. Binding sites of limonene in CBPPs S18

Figure S20. Binding sites of limonene in CBPPs (II) S19

9.-References S19

1.- Materials and Characterization Methods

All of the reagents were obtained from commercial sources and used without further purification unless otherwise indicated. Solvents were dried by standard methods or by elution using a PureSolv Innovative Technology drying system.

Mass spectra were acquired on a linear MALDI TOF/TOF (ULTRAFLEX III BRUKER). The analysis was done using DCTB as a matrix with Positive Reflector model and 355 nm laser NdYAG. The HR-MS analysis was carried out by using an Agilent 1200 Series LC system (equipped with a binary pump, an autosampler, and a column oven) coupled to a 6520 quadrupole-time of flight (QTOF) mass spectrometer. Acetonitrile: water (75:25, v:v) was used as mobile phase at 0.2 mL min^{-1}. The ionization source was an ESI interface working in the positive-ion mode. Nuclear magnetic resonance (NMR) spectra were recorded with a BRUKER AVANCE III HD (Larmor frequencies of 400 and 101 MHz for ^{1}H and, ^{13}C respectively) for liquids and a Bruker AV400 WB spectrometer (Larmor frequencies of 400 and 100 MHz, using 4 mm MAS probes spinning at 10 kHz rate for ^{13}C solid-state MAS-NMR measurements. The ^{13}C CP-MAS spectra were obtained using 3.5 ms contact time and 4 s relaxation time. The number of scans was 1024 of ^{13}C CP-MAS spectra. ATR-FTIR spectra were recorded (cm^{-1}) on a PerkinElmer Spectrum Two spectrometer with a Fourier equipped with a diamond internal element. Specific rotation of the optically active samples was determined on a JASCO P-2000 Polarimeter using sodium lamp (589 nm). Circular Dichroism was performed on a J-815 equipment of Jasco provided with a peltier set at 25ºC with 1.0 cm quartz cells. The microwave used was Discover SP® from CEM Corporation 3100 and/or Monowave 300 from Anton PAAR. Acquisition Gas Chromatographic (GC) was done using KONIK HRGC 5000B; a CP-CHIRASIL-DEX CB varian capillary column (25 m, 0.25 mm, 0.25 µm) and KAP-120212 capillary column (15 m, 0.25 mm, 0.25 µm). Nitrogen adsorption isotherms where measured at 77 K using a Micromeritics ASAP 2020 M and Quantachrome surface and porosity analyzer. Prior to measurement, the samples were degassed for 12 h at 100°C. Specific surface areas were determined by N_2 adsorption-desorption at 77K and the pore distribution by DFT methods.

Fluorescence spectra were recorded on a Varian Cary Eclipse fluorescence spectrophotometer equipped with 1.0 cm quartz cells. The widths of both the excitation slit and the emission slit were set to 5.0 nm. Fluorescence experiments were recorded in acetonitrile solution (for soluble (R)-2Ad-BINOL) and in solid-state and acetonitrile suspension (for CBPPs) at room temperature.

2.-Preparation of BINOL monomers

The synthesis of (*R*)-BINOL precursor (P1) with adamantine groups in the 6,6' position has been already published by our group and the synthetic route shown in scheme 1.[1]

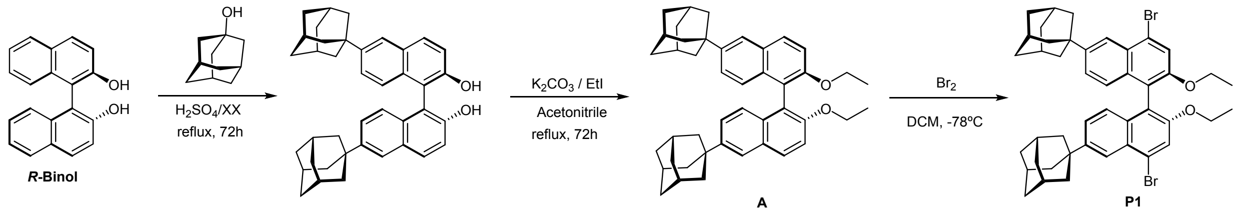

Scheme S1. Synthetic route to obtain monomer **P1**.

Herein, we described the analogous (*S*)-BINOL precursor with *tert*-butyl groups in the 6,6' position and the route is showed in scheme 2:

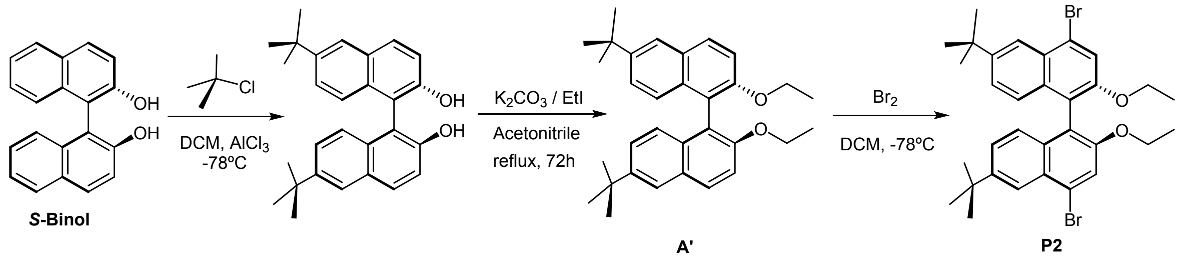

Scheme S2. Synthetic route to obtain monomer **P2**.

(*S*)-6,6'-di-*tert*-butyl-2,2'-diethoxy-1,1'-binaphthalene (A')

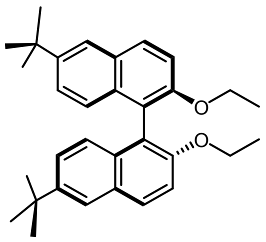

(*S*)-6,6'-Di-*tert*-butyl-1,1'-binaphthyl-diol**[1,2]** (2.68 g, 4.83 mmol, 1.0 eq.) was dissolved in acetonitrile (40 mL) and then K_2CO_3 (2.67 g, 19.32 mmol, 4.0 eq.) and ethyl iodide (970 μL, 12.08 mmol, 2.50 eq.) were added. The reaction mixture was refluxed during 72 hours. After complete conversion (monitored by TLC heptane: AcOEt 3:1), it was extracted with H_2O and toluene. The organic layer was washed two times with H_2O and one time with brine. Finally, it was dried over $MgSO_4$ and the solvent was removed at low pressure to give a yellow solid. **Yield** (99 %); **mp:** 132-134 ºC. **^{1}H NMR (400 MHz, $CDCl_3$) δ (ppm):** 7.93-788 (m, 1H), 7.78 (m, 1H), 7.40 (d, J = 9.0 Hz, 1H), 7.31 (d, J = 2.1 Hz, 1H), 7.10 (dt, J = 8.9 Hz, 1H), 4.04 (q, J = 7.0 Hz, 2H), 1.38 (s, 9H), 1.07 (t, J = 7.0 Hz, 3H), **^{13}C NMR (101 MHz, $CDCl_3$) δ (ppm):** 153.8, 145.9, 132.4, 129.1, 128.9, 125.3, 125.0, 122.6, 120.4, 115.4, 65.2, 34.5, 31.0, 15.0. **HRMS (ESI+)**. calcd for

$[C_{32}H_{38}O_2]$ 454.29, found: 454.3811 **FT-IR ν(KBr, cm^{-1}):** 3436, 2975, 2902, 2847, 1704, 1593, 1471, 1449, 1340, 1314, 1278, 1234, 1178, 1140, 1114, 1099, 1054, 979, 944, 897, 809, 699**.**

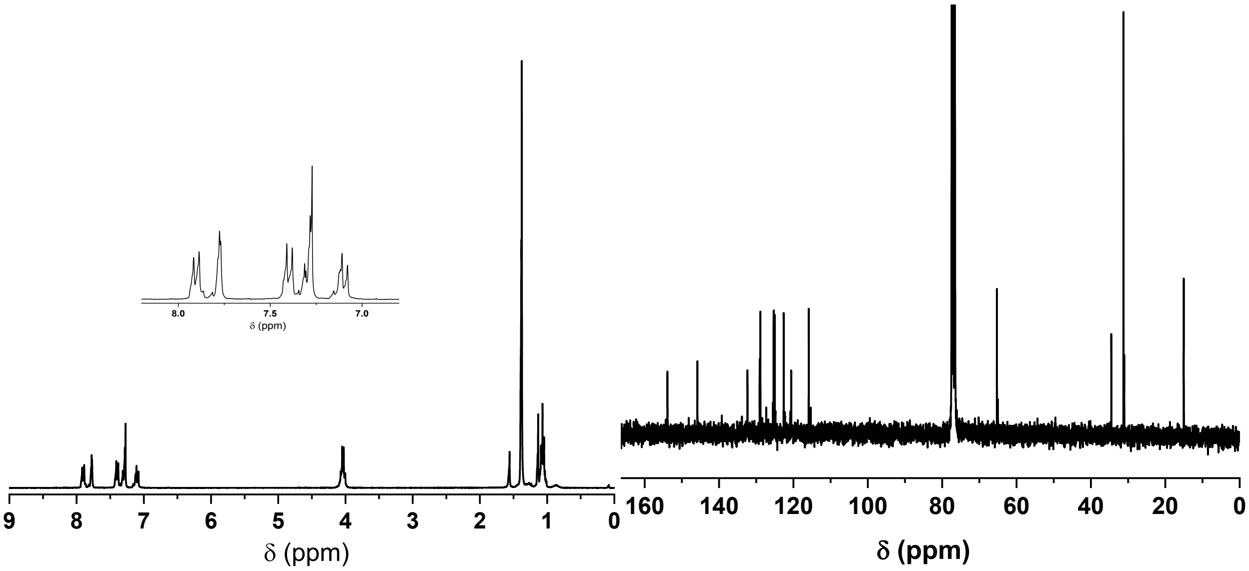

Figure S1. ^{1}H-NMR (left) and ^{13}C-NMR (right) of **A'**.

(*S*)-4,4'-di-bromo-6,6'-di-*tert*-butyl-2,2'-diethoxy-1,1'-binaphthalene (P2)

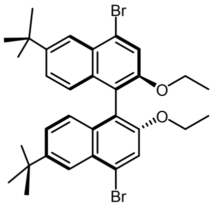

The reaction was done in darkness to prevent the alkylation over aliphatic groups. **A'** (600 mg, 0.98 mmol, 1.0 eq.) was dissolved in DCM (24 mL) and it was cooled to -78 ºC. A solution of Br_2 (135 µL, 2.35 mmol, 2.40 eq.) in DCM (1.5 mL) was added dropwise during 5 minutes. It was maintained for 6 hours and was monitored by TLC using Hep/AcOEt (10:1) as eluent. Sodium bisulfite (10 mL, 20 % w/v) was added to remove the Br_2 excess and it was extracted with DCM. The organic layer was washed two times with H_2O and onetime with brine. Finally, it was dried over $MgSO_4$ and the solvent was removed at low pressure to give a pale yellow solid. **[α]$_{25}^{D}$**= -21.6 (c, 0.1). **Yield** (99 %); **mp:** 307-308 ºC. **^{1}H NMR (300 MHz, CDCl$_3$) δ (ppm)**: 8.16(s, 1H), 7.71 (s, 1H), 7.35-7.31 (d, J = 8.9 Hz, 1H), 7.08-7.05(d, J = 8.9 Hz, 1H), 4.02 (q, J = 7.0 Hz, 2H), 1.40 (m, 9H), 1.07 (m, 3H). **^{13}C NMR (101 MHz, CDCl$_3$) δ (ppm):** 153.46, 147.61, 132.85, 127.44, 126.70, 126.00, 123.63, 122.06, 121.89, 119.79, 119.36, 65.28, 34.89, 31.17 14.91. **HRMS (ESI+)**: calcd for $[C_{32}H_{36}Br_2O_2]$ 610.1082, found: 610.1093.

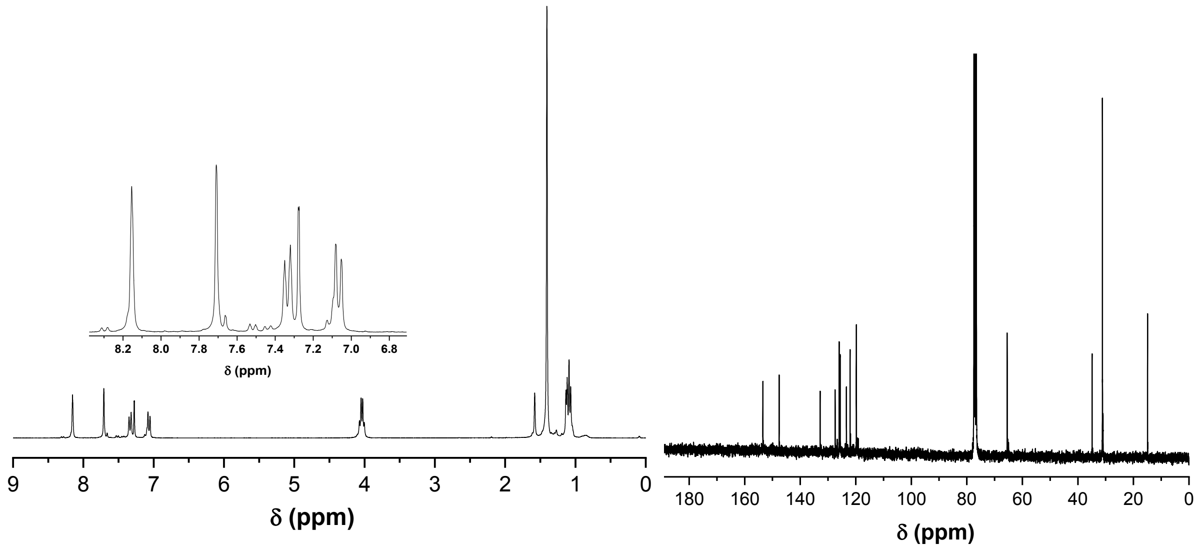

Figure S2. ^{1}H-NMR (left) and ^{13}C-NMR (right) of precursor **P2**.

3.-Preparation of BINOL-based polymers (CBPPs)

Preparation of CBPPs-OEt via Suzuki-Miyaura coupling:

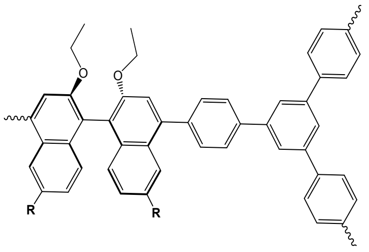

General Method: BINOL monomer **(P1)** (0.652 mmol, 1.5 eq.), 1,3,5-triphenylbenzene-4',4'',4'''-triboronic acid[3] **(M1)** (191 mg, 0.436 mmol, 1.0 eq.), K_2CO_3 (2 mL, 2 M, 4.20 eq.) and dry THF (4.5 mL) were introduced in a sealed tube and deaerated with argon for 15 minutes. After that, catalyst Pd(dppf)Cl_2 (2.2 mg, 19.6 µmol, 3 %) was added. The reaction was stirred overnight at 100ºC. The resulting solid was filtered and thoroughly washed with H_2O. The solid was stirred with a mixture of acetone-H_2O and KCN overnight to remove the Pd(0) residues. Then, it was filtered and dried to obtain the final product.

Preparation of CBPPs-OEt vía Sonogashira-Hagihara coupling

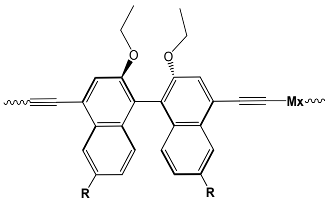

General Method**:** BINOL monomer **(P2)** (120 mg, 0.156 mmol, 1.0 eq.), alkyne (**M2**, **M3**, 0.66 eq. or **M4** 0.5 eq.), 2 mL of DIPA and dry DMF (4.0 mL) were introduced in a sealed tube and deaerated with argon for 15 minutes. After that, catalyst Pd(PPh_3)$_4$ (9.8 µmol, 3 %) and CuI (5 µmol, 1.5 %) were added. The reaction was stirred overnight at 100ºC. The resulting solid was filtered and thoroughly washed with H_2O. The solid was stirred with a mixture of acetone-H_2O and KCN overnight to remove the Pd(0) species. Then, it was filtered and dried to obtain the final product.

Preparation of CBPPs-OH

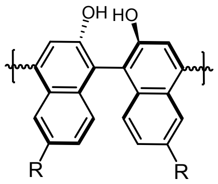

General deprotection procedure: An excess of BBr_3 in DCM (10 mL per 100 mg of polymer) was added at -78 ºC to a suspension of the CBPP and stirred for 2 hours; then the mixture was heated to room temperature and stirred two days at said temperature. To quench the reaction, a saturated aqueous solution of $NaHCO_3$ (5 mL) was added and the mixture was stirred for 2 hours, the resulting polymer was filtered and exhaustively washed with water, subsequently the polymer was stirred in warm methanol for two additional hours. Finally, the solid was filtered and washed with methanol, acetone and diethyl ether.

4.- Characterization of CBPPs

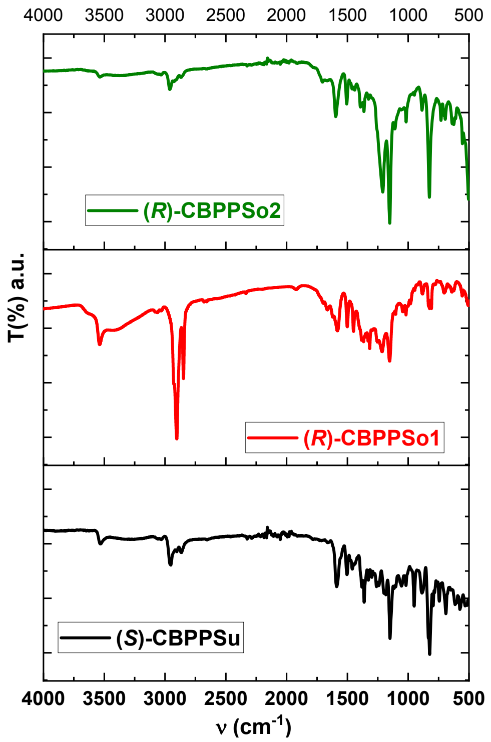

Figure S3. ATR-IR spectra.

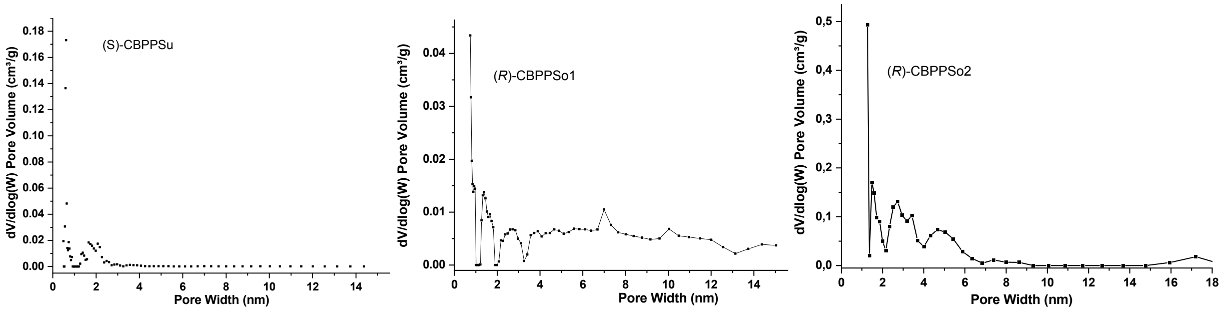

Figure S4. Pore distribution by DFT method.

5.- Uv-vis and fluorescence emission spectra of (*R*)-2Ad-BINOL and CBPPs-OH

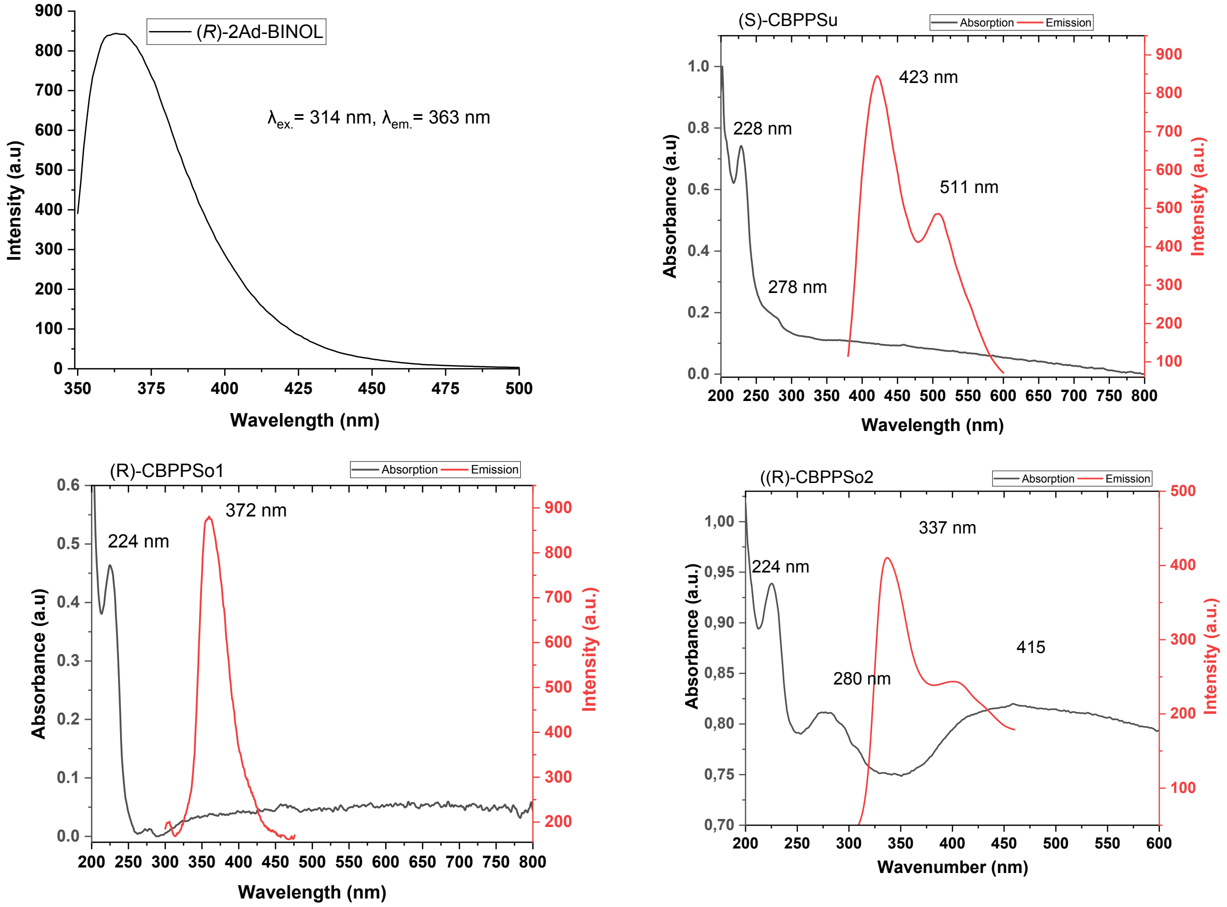

Figure S5. Emission spectra of (*R*)-2Ad-BINOL (c = 10^{-4} M, in acetonitrile solution) (λ_{ex} = 314 nm, λ_{em} = 363 nm (359 nm, lit. for OEt-BINOL)[4] and CBPPs (1 mg) in acetonitrile suspension (4 mL).

6.- UV/vis absorption spectrum of chiral analytes

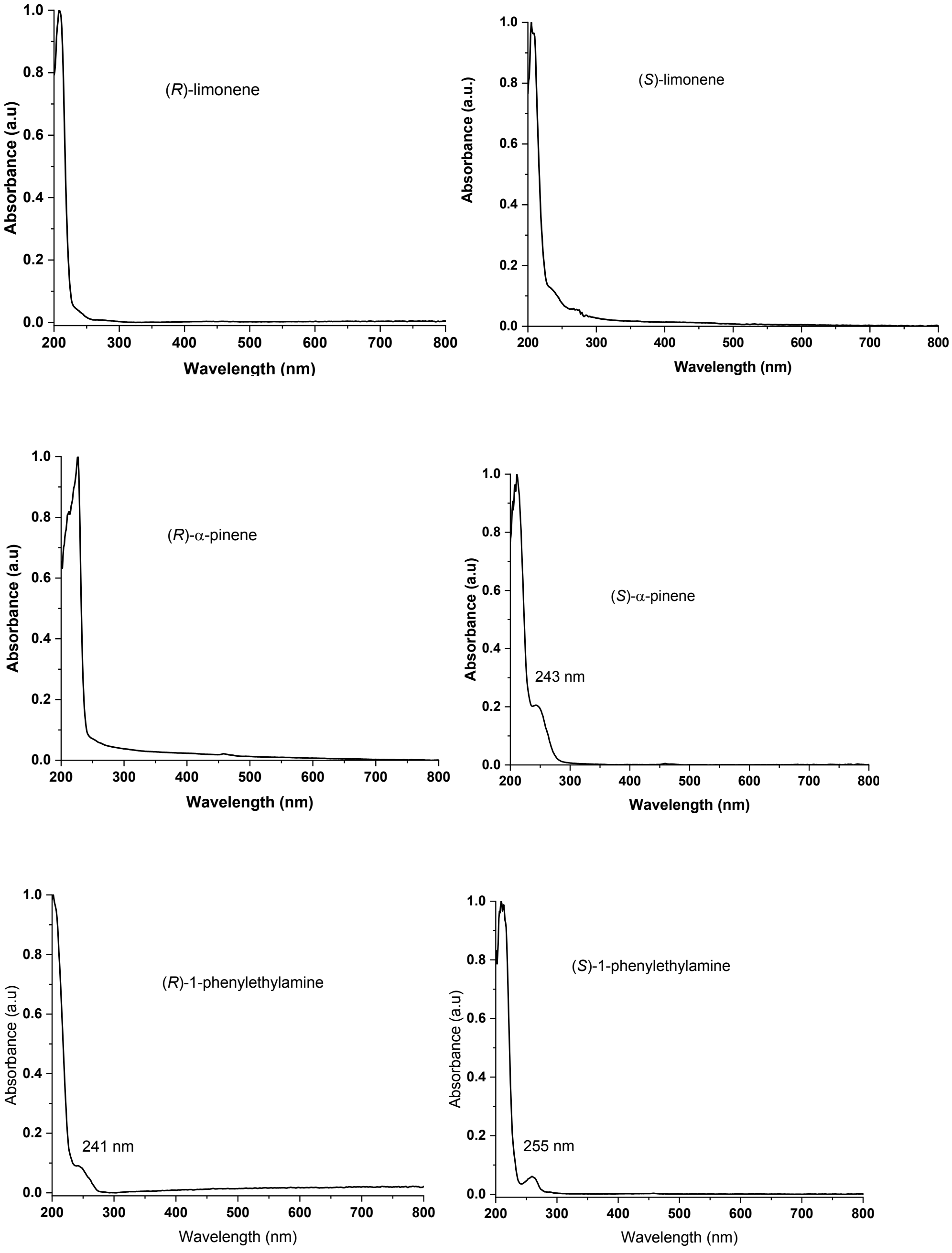

Figure S6. Normalized UV/vis absorption spectra of analytes as a solution ($1x10^{-3}$ mM) in acetonitrile.

7.- General procedures and results for fluorescence sensing

Procedures for quenching measurements

All materials were soaked in deoxygenated acetonitrile to remove any solvent remaining in the porous. Then, the solids were dried under vacuum and mechanically grinded with an agate mortar and pestle. Exactly 1.0 mg of the corresponding CBPPs-OH was placed in a quartz cuvette with 4 mL of acetonitrile leading a cloudy dark suspension. Moreover, 0.5 M enantiopure solutions of quenchers were prepared.

Stern-Volmer plot measurements

Fluorimeter instrumental parameters were set as follows: slit and bandwidths = 5 nm; rate = 600 nm/min. The voltage was adjusted to achieve a good signal to noise without saturation. For (*S*)-CBPPSu: excitation λ = 278 nm, emission λ = 424 nm; for (*R*)-CBPPSo1 excitation λ = 270 nm, emission λ = 372 nm and for (*R*)-CBPPSo2 excitation λ = 280 nm and λ_{em} = 337 nm. A careful reading was performed before the addition of quencher solutions (to obtain I_0 under identical conditions for normalization) and again after each addition of the quencher to the corresponding suspension of the CBPPs-OH in acetonitrile. Samples were allowed to stir for three minutes after addition of each quencher to give enough time for the diffusion of quencher through the CBPPs-OH porous framework. An average intensity was then taken from 5 measurements, after each addition, and used as the intensity for that given quencher concentration.

The fuorescence quenching efficiency is determined by monitoring changes in fluorescence band and is associated to the Stern–Volmer constant (Ksv):

Stern–Volmer equation: $I_0/I = 1 + Ksv[Q]$ with I_0, I as fluorescence band intensity of CBPP without and with analyte respectively, [Q] is the concentration of quencher.[5]

Fluorescence Sensing experiments

7.1 Quenchers (*R*)-limonene and (*S*)-limonene

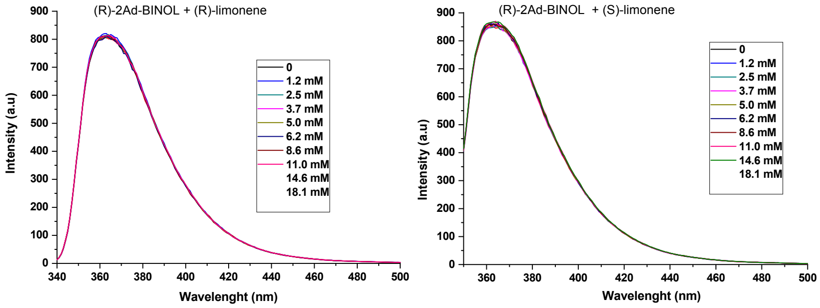

Figure S7. Fluorescence quenching of (*R*)-2Ad-BINOL (λ_{ex} = 314 nm) in acetonitrile (4 mL) upon titration with (*R*)-limonene (left) and (*S*)-limonene (rigth) quenchers.

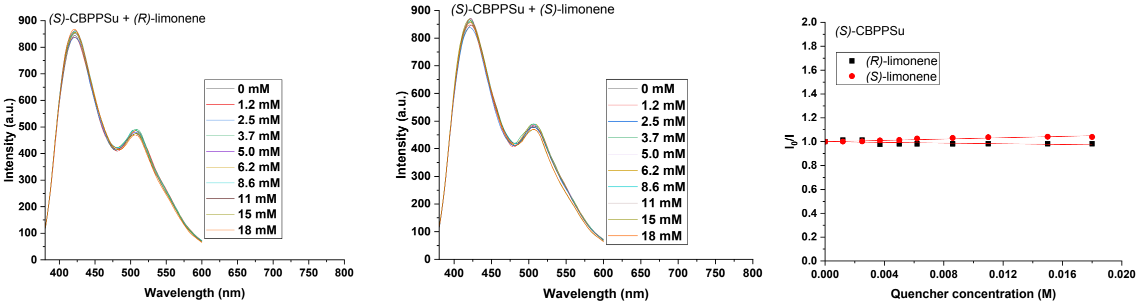

Figure S8. Fluorescence quenching of (S)-CBPPSu (λ_{ex} = 278 nm) (1 mg) in acetonitrile (4 mL) upon titration with (*R*)-limonene (left) and (*S*)-limonene (middle) quenchers and the Stern−Völmer plot of titration (rigth).

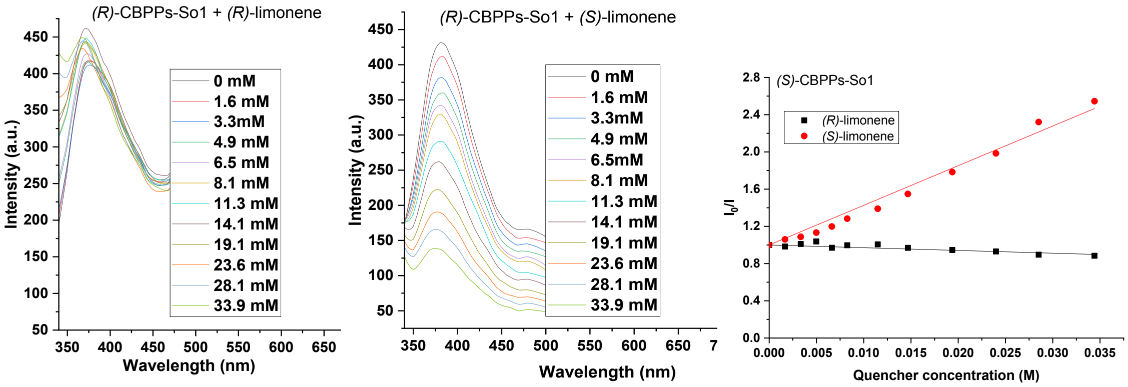

Figure S9. Fluorescence quenching of (*R*)-CBPPSo1 (λ_{ex} = 270 nm) (1 mg) in acetonitrile (4 mL) upon titration with (*R*)-limonene (left) and (*S*)-limonene (middle) quenchers and the Stern−Völmer plot of titration(rigth).

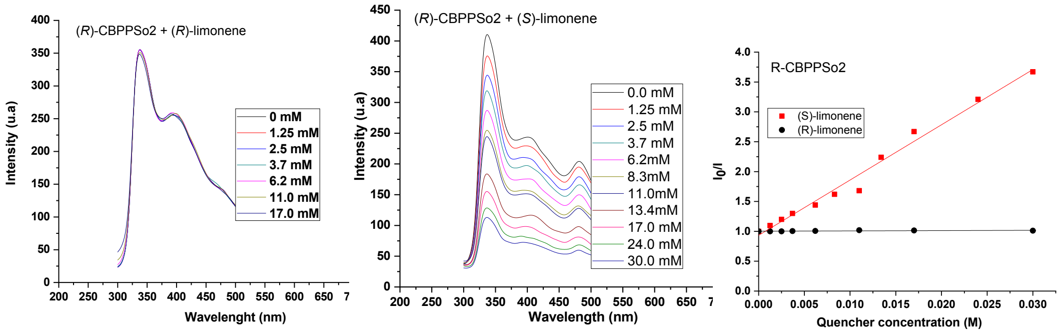

Figure S10. Fluorescence quenching of (*R*)-CBPPSo2 (λ_{ex} = 280 nm) (1 mg) in acetonitrile (4 mL) upon titration with (*R*)-limonene (left) and (*S*)-limonene (middle) quenchers and the Stern−Völmer plot of titration (rigth).

7.2.- - Quenchers (*R*)-Pinene and (*S*)-Pinene

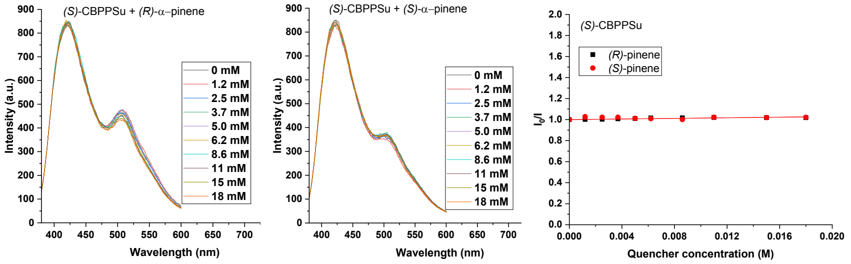

Figure S11. Fluorescence quenching of (*R*)-CBPPSu (λ_{ex} = 278 nm) (1 mg) in acetonitrile (4 mL) upon titration with (*R*)-α-Pinene (left) and (*S*)-α-Pinene (middle) quenchers and the Stern−Völmer plot of titration (rigth).

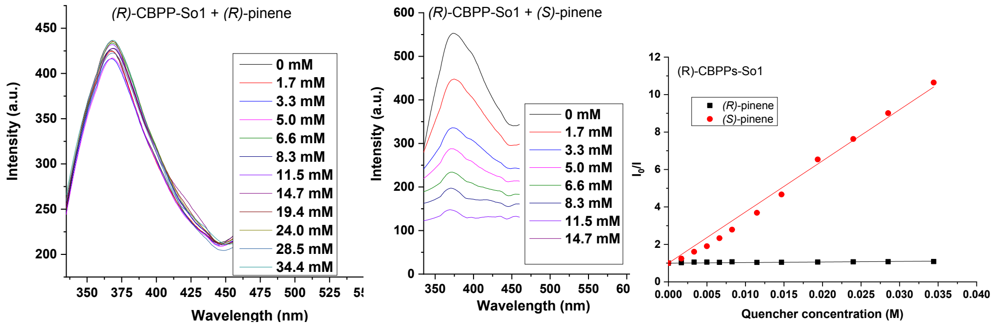

Figure S12. Fluorescence quenching of (*R*)-CBPPSo1 (λ_{ex} = 270 nm) (1 mg) in acetonitrile (4 mL) upon titration with (*R*)-α-Pinene (left) and (*S*)-α-Pinene (middle) quenchers and the Stern−Völmer plot of titration (rigth).

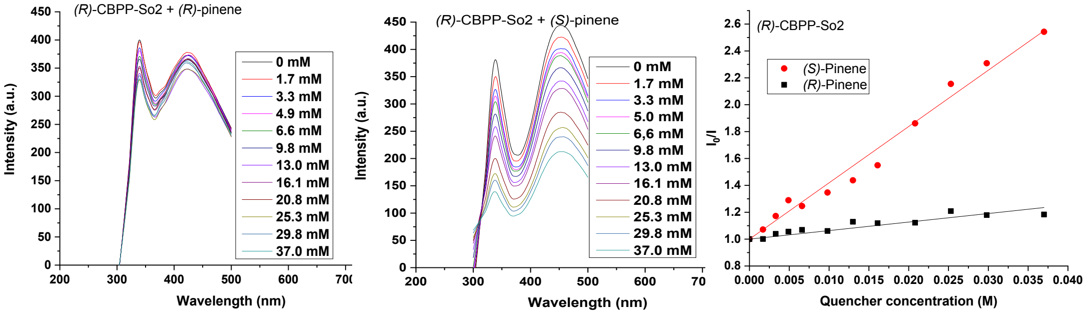

Figure S13. Fluorescence quenching of (*R*)-CBPPSo2 (λ_{ex} = 280 nm) (1 mg) in acetonitrile (3 mL) upon titration with (*R*)-α-Pinene (left) and (*S*)-α-Pinene (middle) quenchers and the Stern−Völmer plot of titration (rigth).

7.3.- Quenchers: (*R*)-1-phenylethylamine and (*S*)-1-phenylethylamine

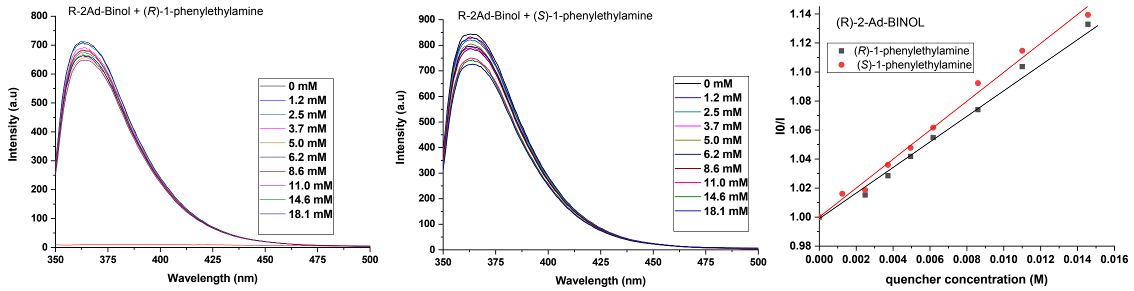

Figure S14. Fluorescence quenching of (*R*)-2-Ad-BINOL (λ_{ex} = 314 nm) in acetonitrile upon titration with (*R*)-1-phenylethylamine (left) and (*S*)-1-phenylethylamine (middle) quenchers and the Stern−Völmer plot of titration (rigth) (c = 10^{-4} M).

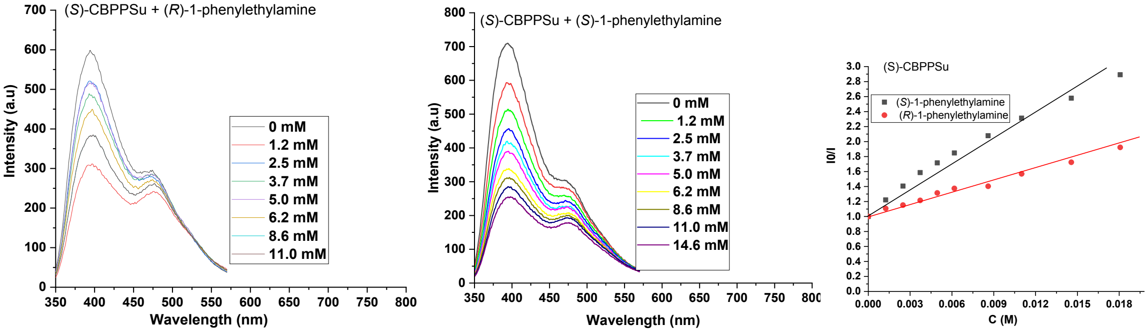

Figure S15. Fluorescence quenching of (*S*)-CBPPSu (λ_{ex} = 278 nm) (1 mg) in acetonitrile (4 mL) upon titration with (*R*)-1-phenylethylamine (left) and (*S*)-1-phenylethylamine (middle) quenchers (up) and the Stern−Völmer plot of titration (rigth).

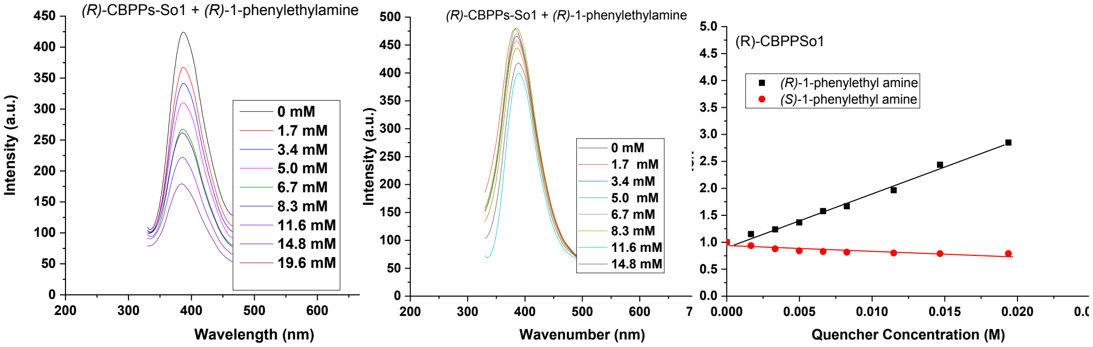

Figure S16. Fluorescence quenching of (*R*)-CBPPSo1 (λ_{ex} = 270 nm) (1 mg) in acetonitrile (4 mL) upon titration with (*R*)-1-phenylethylamine (left) and (*S*)-1-phenylethylamine (middle) quenchers and the Stern−Völmer plot of titration(rigth)

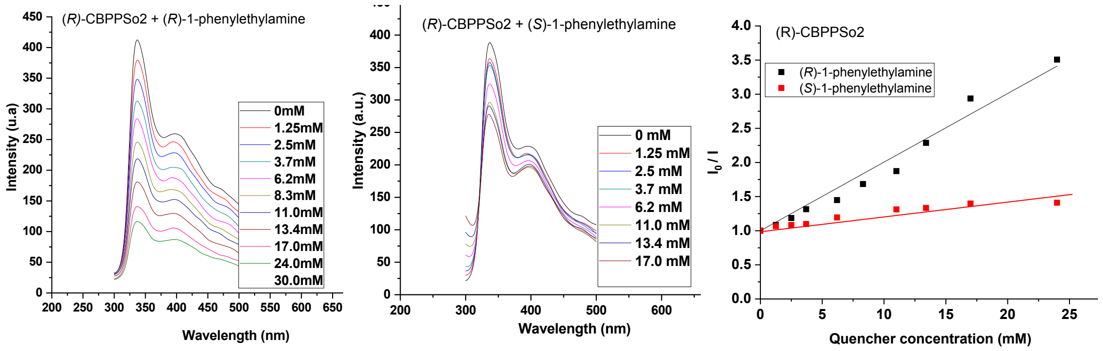

Figure S17. Fluorescence quenching of (*R*)-CBPPSo2 (λ_{ex} = 280 nm) (1 mg) in acetonitrile (4 mL) upon titration with (*R*)-1-phenylethylamine (left) and (*S*)-1-phenylethylamine (middle) quenchers and the Stern−Völmer plot of titration(rigth).

8.- Computational simulations

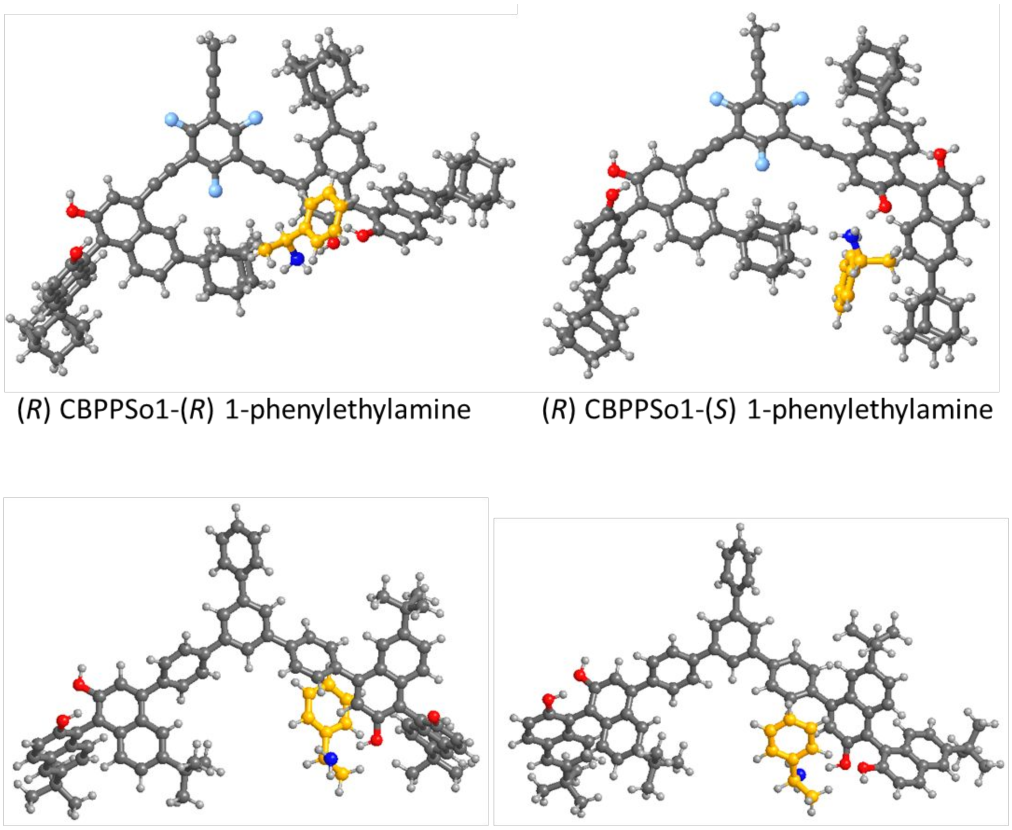

(*R*) CBPPSo1-(*R*) 1-phenylethylamine (*R*) CBPPSo1-(*S*) 1-phenylethylamine

(*S*) CBPPSu-(*R*) 1-phenylethylamine (*S*) CBPPSu-(*S*) 1-phenylethylamine

Figure S18. Optimized geometry of 1-phenylethylamine (in yellow) interacting with (*R*)-CBPPSo1 and (*S*)-CBPPSu polymer models. C: gray; H: white: O: red; N: blue, F: light blue.

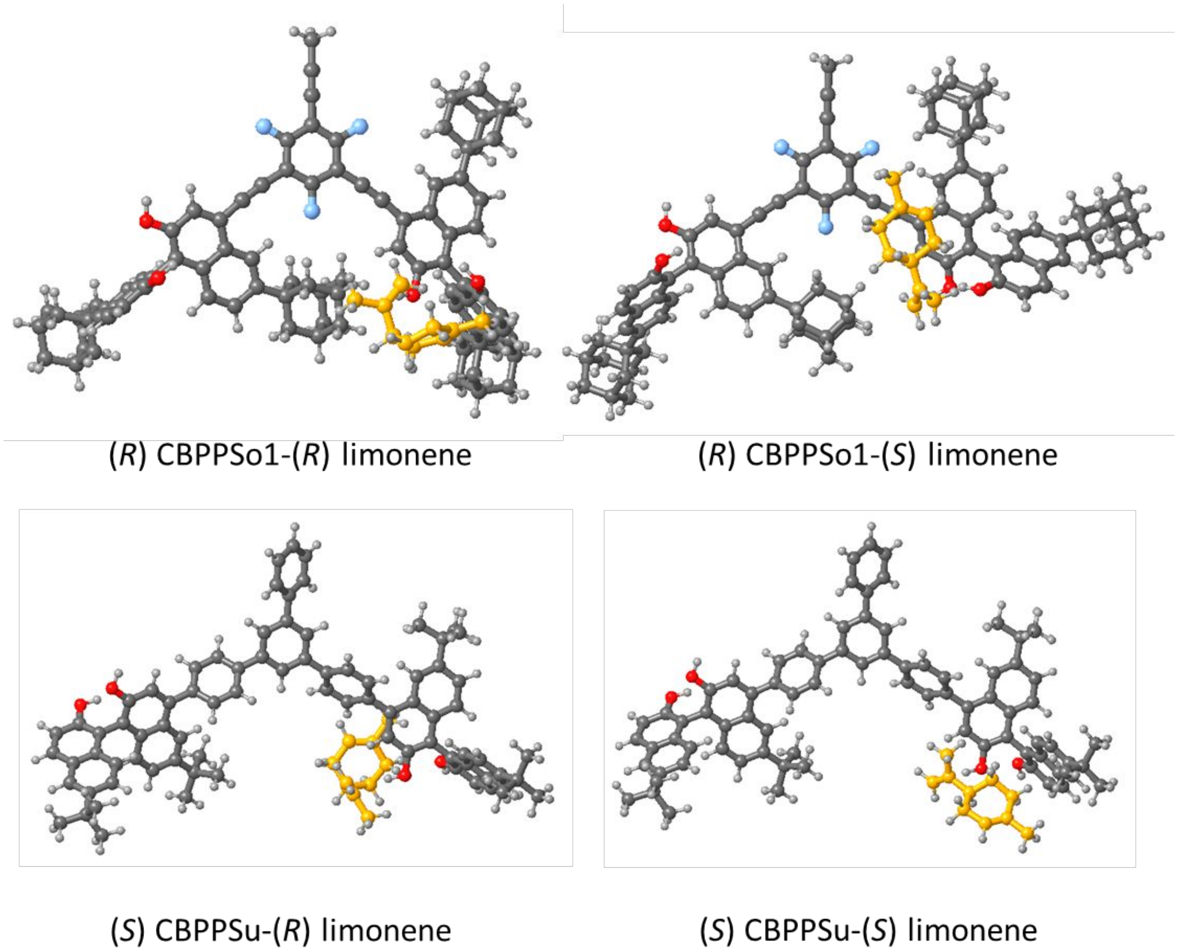

Figure S19. Optimized geometry of limonene (in yellow) interacting with (*R*)-CBPPSo1 and (*S*)-CBPPSu polymer models. C: gray, H: white, O: red, F: light blue.

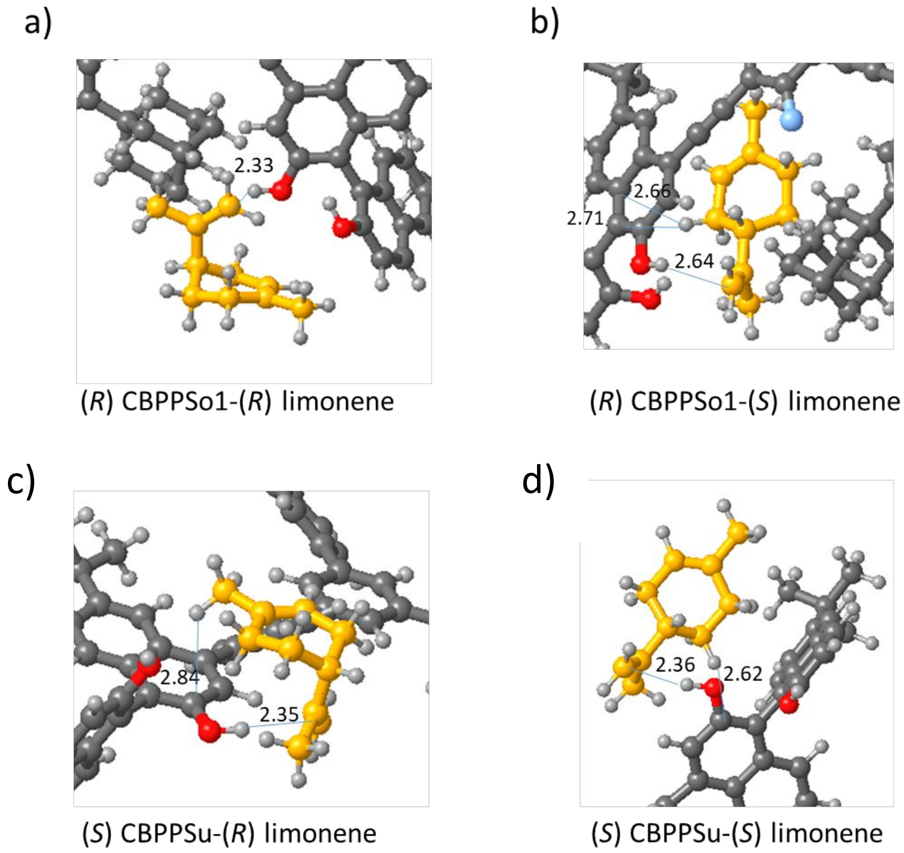

Figure S20. Optimized geometry of limonene (in yellow) interacting with (*R*)-CBPPSo1 (a,b) and (*S*)-CBPPSu (c y d) polymers. Optimized bond lengths in Å. C: gray, H: white, O: red, F: light blue.

9.-References

[1] Navarro, R.; Monterde, C.; Iglesias, M.; Sánchez, F. Readily Available Highly Active [Ti]-Adamantyl-BINOL Catalysts for the Enantioselective Alkylation of Aldehydes *ACS Omega* **2018**, *3,* 1197−1200

[2] Balaraman, E.; Kumara Swamy, K. C. *Tetrahedron Asymm.* **2007**, *18* (17), 2037–2048.

[3] Zhang, Y. B.; Furukawa, H.; Ko, N.; Nie, W.; Park, H. J.; Okajima, S.; Cordova, K. E.; Deng, H.; Kim, J.; Yaghi, O. M. *J. Am. Chem. Soc.* **2015**, *137* (7), 2641–2650

[4] Pu, L. Fluorescence of Organic Molecules in Chiral Recognition. *Chem. Rev.* 2004, 104, 1687–1716.

[5] Wang, Q.; Chen, X.; Tao, L.; Wang, L.; Xiao, D.; Yu, X. Q.; Pu, L. Enantioselective Fluorescent Recognition of Amino Alcohols by a Chiral Tetrahydroxyl 1,1′-Binaphthyl Compound. *J. Org. Chem.* **2007**, *72* (1), 97–101.
